# Peripheral residence of naïve CD4 T cells induces MHC class II-dependent alterations in phenotype and function

**DOI:** 10.1186/s12915-014-0106-0

**Published:** 2014-12-21

**Authors:** Sanket Rane, Rituparna Das, Vidya Ranganathan, Savit Prabhu, Arundhoti Das, Hamid Mattoo, Jeannine Marie Durdik, Anna George, Satyajit Rath, Vineeta Bal

**Affiliations:** National Institute of Immunology, New Delhi, 110067 India; Department of Biological Sciences, University of Arkansas, Fayetteville, Arkansas USA; Current address: Yale Cancer Center, Sterling Hall of Medicine, New Haven, USA; Current address: Division of Genetics & Development, Toronto Western Research Institute, University Health Network, Toronto, Ontario Canada; Current address: Pediatric Biology Centre, Translational Health Sciences and Technology Institute, Gurgaon, India; Current address: MGH Cancer Center, Charlestown, USA

**Keywords:** CD4 T cell, Naïve, Peripheral residence, MHC class II, Th1, Th2

## Abstract

**Background:**

As individual naïve CD4 T lymphocytes circulate in the body after emerging from the thymus, they are likely to have individually varying microenvironmental interactions even in the absence of stimulation via specific target recognition. It is not clear if these interactions result in alterations in their activation, survival and effector programming. Naïve CD4 T cells show unimodal distribution for many phenotypic properties, suggesting that the variation is caused by intrinsic stochasticity, although underlying variation due to subsets created by different histories of microenvironmental interactions remains possible. To explore this possibility, we began examining the phenotype and functionality of naïve CD4 T cells differing in a basic unimodally distributed property, the CD4 levels, as well as the causal origin of these differences.

**Results:**

We examined separated CD4hi and CD4lo subsets of mouse naïve CD4 cells. CD4lo cells were smaller with higher CD5 levels and lower levels of the dual-specific phosphatase (DUSP)6-suppressing micro-RNA miR181a, and responded poorly with more Th2-skewed outcomes. Human naïve CD4lo and CD4hi cells showed similar differences. Naïve CD4lo and CD4hi subsets of thymic single-positive CD4 T cells did not show differences whereas peripheral naïve CD4lo and CD4hi subsets of T cell receptor (TCR)-transgenic T cells did. Adoptive transfer-mediated parking of naïve CD4 cells *in vivo* lowered CD4 levels, increased CD5 and reactive oxygen species (ROS) levels and induced hyporesponsiveness in them, dependent, at least in part, on availability of major histocompatibility complex class II (MHCII) molecules. ROS scavenging or DUSP inhibition ameliorated hyporesponsiveness. Naïve CD4 cells from aged mice showed lower CD4 levels and cell sizes, higher CD5 levels, and hyporesponsiveness and Th2-skewing reversed by DUSP inhibition.

**Conclusions:**

Our data show that, underlying a unimodally distributed property, the CD4 level, there are subsets of naïve CD4 cells that vary in the time spent in the periphery receiving MHCII-mediated signals and show resultant alteration of phenotype and functionality via ROS and DUSP activity. Our findings also suggest the feasibility of potential pharmacological interventions for improved CD4 T cell responses during vaccination of older people via either anti-oxidant or DUSP inhibitor small molecules.

**Electronic supplementary material:**

The online version of this article (doi:10.1186/s12915-014-0106-0) contains supplementary material, which is available to authorized users.

## Background

Peripheral residence of naïve CD4 T cells is marked by many complex microenvironmental interactions. Clonal T cell receptors (TCRs) expressed on individual T cells are likely to have variable avidity for the self-peptide MHC class II (spMHCII) complexes they are positively selected on in the thymus, and this avidity leads to tuning of the response of individual T cells [1-3]. Naïve CD4 T cells survive because they receive tonic signals through these spMHCII complexes [4]. The levels and availability of these spMHCII complexes are variable [5], plausibly leading to variations in the frequencies of tonic signaling for individual CD4 T cells. Notably, TCR-mediated signal sensitivity in mouse T cells is also modulated by miRNA-181a [6], which represses expression of dual-specific phosphatase-6 (DUSP6), a molecule linked to human longevity [7] which suppresses phosphorylation of Erk in human T cells [8]. The levels of CD5, a negative regulator of TCR signaling [9], show modulations correlated with the efficiency of TCR-mediated tonic signaling required for peripheral T cell survival [10-12]. There is also evidence that persistent tonic signaling itself may lead to hyporesponsiveness and reduction in co-receptor levels in naive CD8 T cells [13].

Further, naïve T cells can proliferate in the periphery in a cognate ligand-independent fashion if they are in lymphocyte-depleted environments such as during early fetal-neonatal development in the neonatal mouse or in the aged animal with reduced thymic output [14-17], although the precise functional consequences of such homeostatic proliferation are not yet clear [17,18]. Cytokines such as IL-2, −7, −15 and their receptors have been implicated in peripheral T cell homeostasis and survival [18-20], and common receptor gamma-chain-deficient naive CD4 cells in the periphery are smaller in size [19], consistent with the possibility that cellular metabolism may be a major variable in determining peripheral survival and functionality of naïve T cells [21-23]. Finally, stochasticity in levels of gene expression and responsiveness is expected to mark any apparently homogenous population of cells including T cells [24-29], although it is difficult to distinguish between subset-dependent variations and true statistical noise.

The thymus continuously exports naïve mature T cells to the periphery with declining output due to age-associated involution. Many of these naïve T cells in the periphery remain naïve until their eventual loss from the peripheral pool [30]. Thus, the post-thymic, peripheral residence times of naive T cells at any given point in time would be variable. As thymic output decreases with age, naïve T cells in the periphery also appear to be longer-lived [16-18,31]. Thus, it is likely that the naïve T cells from aged animals will show a longer average peripheral residence time than those from young individuals [32,33], and this may be connected to the functional CD4 T cell defects seen in aged animals [16,34]. While there are some markers to identify recent thymic emigrants [35,36], there are currently no phenotypic modalities to measure peripheral residence time in individual naïve T cells in heterogeneous populations.

On this background, we began looking for variable functional behavior in mouse (and human) peripheral naïve CD4 (NCD4) T cells differing in properties that were apparently unimodally distributed, with the initial expectation that these differences would be stochastic in nature. CD4 was chosen since it shows low variance in its distribution and is uniformly present without major alterations through the entire lifespan of CD4 T cells. We find that the top and bottom decile of CD4 levels in NCD4 T cells define cells that are both phenotypically and functionally different, with NCD4lo cells having smaller sizes, higher CD5 levels, poor responses, lower levels of the DUSP6-suppressing micro-RNA181a, and greater susceptibility to death and to Th2-skewing. However, we find that these differences are not stochastic; they are not found in NCD4lo and NCD4hi T cells in the thymus, but they persist even when the TCR repertoire of the CD4 T cell population is homogenized as in NCD4 T cells from TCR-transgenic mice, and they can be induced by exposure *in vivo* to MHCII-mediated tonic signals during a few days of peripheral residence, indicating that unimodal distribution of a variable does not necessarily mean that the variability is stochastic. DUSP and reactive oxygen species (ROS) appear to mediate the MHCII-induced hyporesponsiveness of NCD4 T cells, since ROS scavenging or DUSP inhibition ameliorate it. Finally, consistent with the greater average time of peripheral residence of NCD4 T cells in aged animals [16], we find that the properties found in NCD4lo T cells from young mice are also found in the NCD4 T cells of aged mice, making ROS and DUSP potential targets for intervention for successful vaccination in the older population.

## Results

### Despite unimodal distribution, CD4 levels on naïve CD4 T cells are correlated with responsiveness

**Figure 1:**
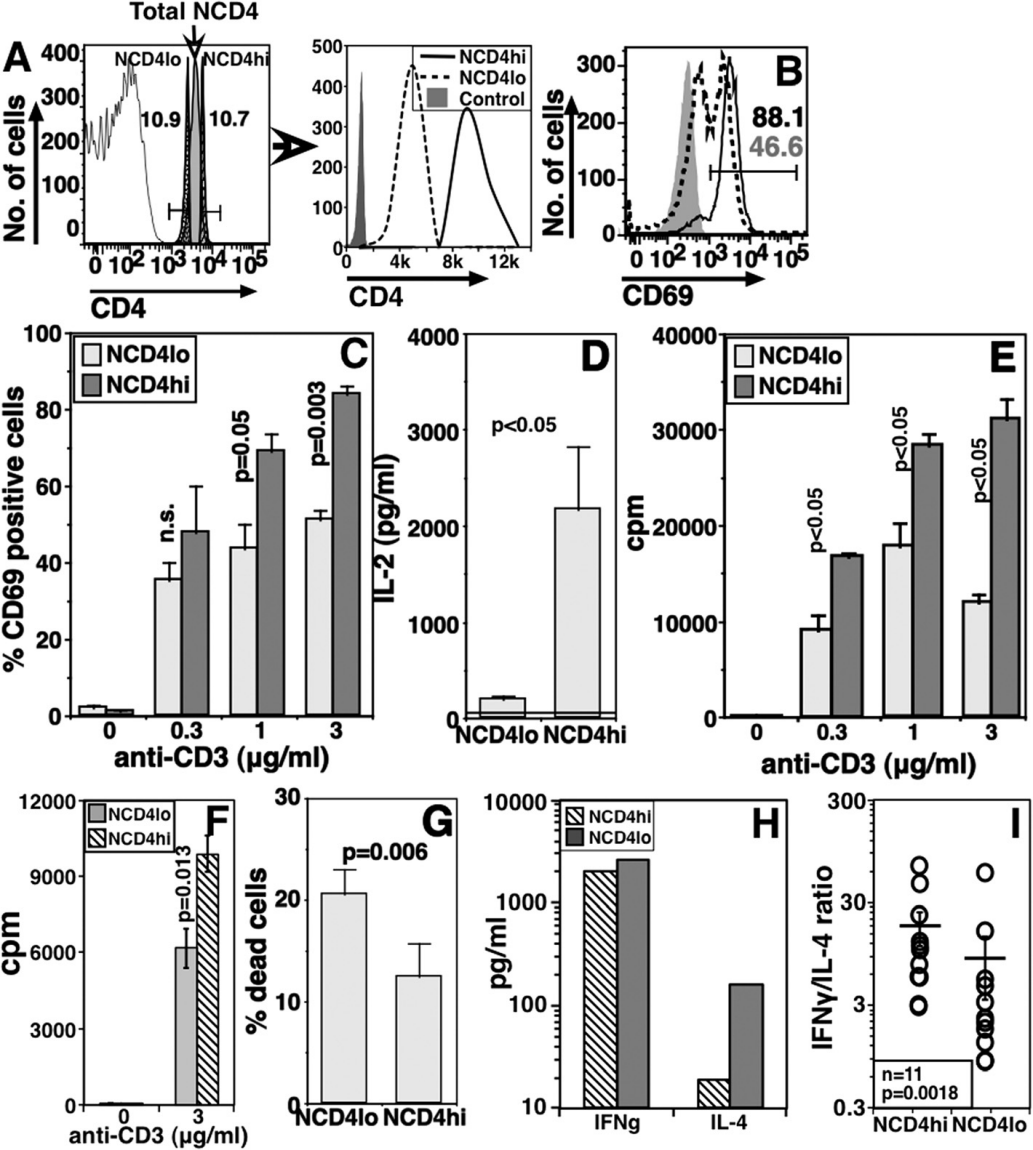
**Despite unimodal distribution, CD4 levels on naïve CD4 T cells are correlated with responsiveness. A**. Gating strategy used to sort NCD4lo and NCD4hi cells from six- to eight-week-old mice. Right panel shows sort profile for NCD4hi and NCD4lo cells. **B**. CD69 expression on anti-CD3 + anti-CD28 (3 μg/ml each) stimulated NCD4lo and NCD4hi cells 16 hours post-activation. Numbers indicate representative proportions of CD69+ cells in NCD4hi (black) and NCD4lo (grey) cells. **C**. Proportions of CD69+ cells in a dose–response curve with 3 μg/ml of anti-CD28 and titrating anti-CD3 concentrations as shown. (Mean ± SE, n = 4; n.s.: not significant). **D**. Amount of IL-2 detected 48 hours post-stimulation with anti-CD3 + anti-CD28 in culture supernatants. (Mean ± SE, n = 3; Background values shown as a line). **E**. 3H-Thymidine incorporation assay to measure proliferation 60 hours post stimulation with anti-CD3 + anti-CD28. (Mean ± SE of triplicate cultures, 1 of >7 experiments). **F**. 3H-Thymidine incorporation assay on sorted NCD4hi and NCD4lo cells cultured *in vitro* with IL-2 for 24 hours prior to stimulation with anti-CD3 + anti-CD28 for 60 hours. (Mean ± SE of triplicate cultures, one of three experiments). **G**. Proportion of trypan blue-positive cells 16 hours post-activation (Mean ± SE, n = 6, data representative of four experiments). **H**. Cytokine levels in supernatants of *in vitro* primed and recalled NCD4hi and NCD4lo cells. **I**. IFNγ/IL-4 ratios and mean ± SE calculated based on absorbance values. SE, standard error.

NCD4 cells show unimodal distribution of CD4 levels. To examine whether this apparently homogenous population has any functional consequences, we sorted mouse splenic NCD4 cells (CD4 + CD25-CD44-CD62L+) into the brightest (NCD4hi) and dullest (NCD4lo) deciles of CD4 levels (Figure 1A). There was no overlap in the CD4 levels of these sorted populations, which typically differed by approximately two-fold (Figure 1A). We next characterized these sorted NCD4hi and NCD4lo cells in functional terms. Purified cells were activated with plate-coated anti-CD3 + anti-CD28 monoclonal antibodies (mAbs) for 18 hours and the frequency of cells showing induction of CD69 as an early activation marker was estimated. Smaller proportions of NCD4lo cells than of NCD4hi cells expressed CD69 at multiple anti-CD3 concentrations (Figures 1B and C). Further, NCD4lo cells produced less IL-2 at 48 hours (Figure 1D) and incorporated less (3H)-thymidine at 60 hours post-activation (Figure 1E). Poor proliferation of activated NCD4lo cells was also confirmed in a carboxyfluorescein succinimidyl ester (CFSE) dilution assay (see Additional file 1: Figure S1A-B). We examined the possibility that anti-CD4 antibody bound during sorting signals differentially to the NCD4hi and NCD4lo cells, by resting the sorted cells for 24 hours in IL-2 before stimulating them with anti-CD3 + anti-CD28 for 48 hours. The difference in their proliferative responses persisted, indicating that it was not related to any anti-CD4-mediated signaling artifact (Figure 1F).

Since IL-2 induction was different between NCD4lo and NCD4hi cells, we tested if exogenous IL-2 supplementation could abolish the difference. When exogenous IL-2 (10 U/ml) was added during activation, NCD4lo cells showed some improvement in proliferative responses at lower anti-CD3 concentration, but at no point did their responses reach levels achieved by NCD4hi cells (see Additional file 1: Figure S1C), suggesting that, while differential IL-2 production may contribute to the difference, it was unlikely to represent the sole explanation. There was more prominent activation-induced cell death in NCD4lo cells at 24 hours post-activation, as scored by trypan blue staining, than in NCD4hi cells (Figure 1G).

We also tested the ability of sorted NCD4hi and NCD4lo cells to differentiate *in vitro* in response to non-polarizing activating conditions (described in detail in Methods). Supernatants from cultures of NCD4hi and NCD4lo cells primed *in vitro* with anti-CD3 + anti-CD28 for three days and then restimulated with titrating doses of anti-CD3 were analyzed for IFNγ and IL-4. Effector cells generated from NCD4lo cells produced more IL-4 as compared to those from NCD4hi cells (Figure 1H). The relative Th1/Th2 dominance, indicated by the IFNγ/IL-4 ratio, was significantly lower in differentiated NCD4lo cells (Figure 1I).

### NCD4lo cells show complex phenotypic modifications, but no differences in proximal signaling events

**Figure 2:**
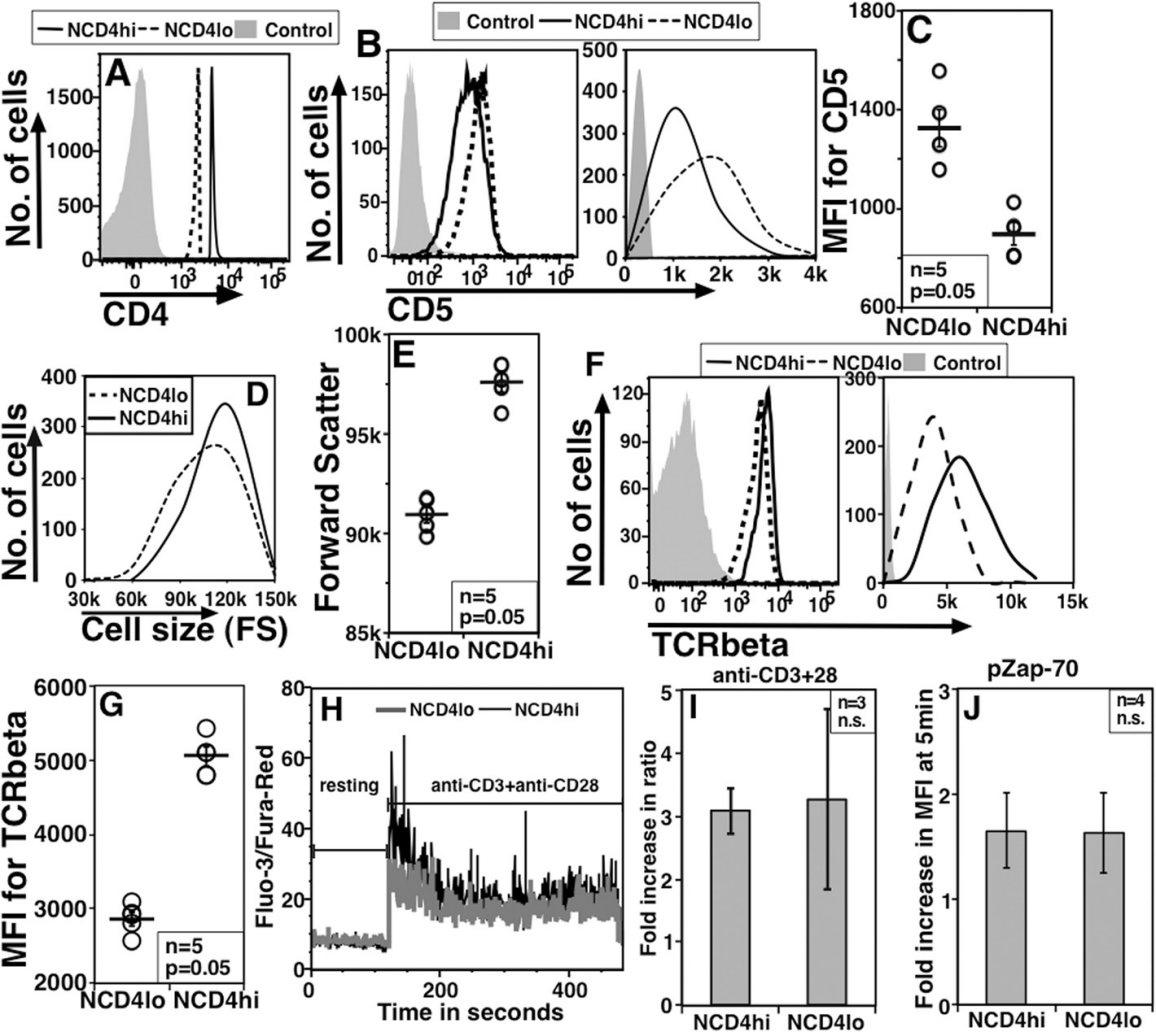
**NCD4lo cells show higher CD5 levels but no difference in proximal signaling. A**. *Ex vivo* analysis of stained splenic cells to show gated NCD4lo and NCD4hi cells. **B**. Comparison of CD5 levels as log (left) and linear (right) plots between NCD4hi and NCD4lo cells. **C**. CD5 MFI values for NCD4hi and NCD4lo cells (Mean ± SE, one of three experiments). **D**. Comparison of forward scatter between NCD4hi and NCD4lo cells. **E**. Forward scatter values on NCD4hi and NCD4lo cells (Mean ± SE, one of five experiments). **F**. Comparison of TCRβ levels as log (left) and linear (right) plots between NCD4hi and NCD4lo cells. **G**. TCRβ MFI values for NCD4hi and NCD4lo cells (Mean ± SE, one of three experiments). **H**. Representative profile of Fluo-3/Fura-Red ratio in NCD4hi and NCD4lo cells at rest and post-activation. **I**. Pooled data from independent mouse spleens showing increase in Fluo-3/Fura-Red ratio with anti-CD3 (10 μg/ml) + anti-CD28 (3 μg/ml) treatment **J**. Pooled data for fold increase over background in pZap-70 staining MFI in NCD4hi and NCD4lo cells activated with anti-CD3 + anti-CD28, mean ± SE. MFI, mean fluorescence intensity; NCD4, naïve CD4 T cells; SE, standard error; TCR, T cell receptor.

CD5 is a negative regulator of TCR signaling [10] and CD5 expression has been inversely correlated with T cell responsiveness [37]. We, therefore, tested the possibility that lower CD4 levels might correlate with higher CD5 expression. NCD4hi and NCD4lo cells from young B6 mice (Figure 2A) were tested for the levels of CD5. When gated NCD4hi and NCD4lo cells were analyzed for CD5 expression, it was apparent that NCD4lo cells had higher CD5 levels (Figure 2B, log plot on left, linear on right) in multiple mice analyzed (Figure 2C). This was despite the finding that NCD4hi cells were somewhat larger than NCD4lo cells (Figures 2D and E). We also examined the levels of a number of other molecular markers in these populations. NCD4lo cells showed lower TCRβ (Figure 2F, log and linear plots; and 2G) levels that NCD4hi cells, although both NCD4lo and NCD4hi cells were confirmed to be conventional CD4 T cells expressing TCRα/β. NCD4lo cells also expressed somewhat lower levels of MHC class I and CD2, but higher levels of CD54 (see Additional file 2: Figure S2A and S2B) in multiple mice examined. Another feature tested based on differences in cell size between NCD4lo and NCD4hi cells was mitochondrial mass and potential using indicator dyes, Mitotracker Green (MG) and Mitotracker Red (MR) [38,39]. However, NCD4lo cells did not show any striking differences from NCD4hi cells in mitochondrial content or potential (see Additional file 2: Figure S2C to S2E). These data indicated that the phenotypic differences between NCD4lo and NCD4hi cells were likely to be complexly regulated.

Since far-downstream proliferative outcomes of TCR activation differed between NCD4lo and NCD4hi cells, we next examined proximal signaling events after ligation with anti-CD3 + anti-CD28. Activation-induced calcium flux (as measured by an increase in Fluo-3/Fura-Red ratios) was indistinguishable between NCD4hi and NCD4lo cells (Figure 2H, 2I and Additional file 2: Figure S2F). Similarly, total Zap-70 levels were comparable between NCD4hi and NCD4lo cells (see Additional file 2: Figure S2G, left panel), and the induction of Zap-70 phosphorylation upon activation for five minutes did not differ between NCD4lo and NCD4hi cells (Figure 2J and Additional file 2: Figure S2G, middle and right panels as log and linear plots). Similarly, total Lck levels were comparable in NCD4lo and NCD4hi cells, and the extent of p-Lck induction at five minutes post-activation was similar between them (see Additional file 2: Figure S2H). Together, these data suggest that proximal signaling is not different between NCD4hi and NCD4lo cells, and that the differences between them may lie further downstream.

### Hypo-responsiveness associated with NCD4lo cells develops exclusively in the periphery and is also seen in monoclonal T cells

**Figure 3:**
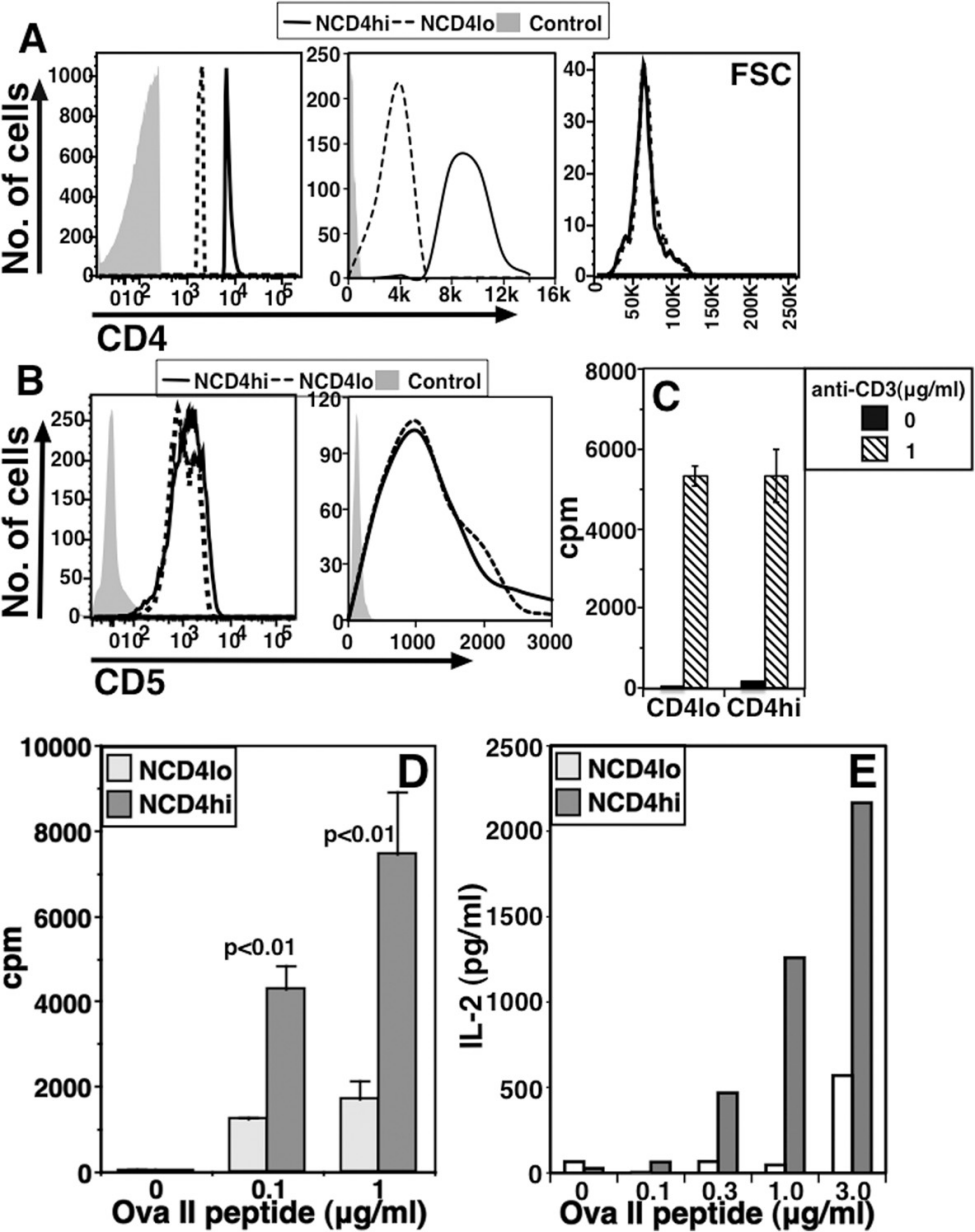
**Hypo-responsiveness associated with NCD4lo cells develops exclusively in periphery and is also seen in monoclonal T cells. A**. *Ex vivo* analysis of stained thymic cells to show single positive mature CD4lo and CD4hi cells (log and linear plots), and corresponding forward scatter (right) (one of three experiments). **B**. Levels of CD5 in log (left) and linear (right) scale from the same experiment as in A. **C**. 3H-Thymidine incorporation assay to measure proliferation of sorted thymic single positive mature CD4lo and CD4hi cells 60 hours post-activation with anti-CD3 (1 μg/ml) and anti-CD28 (3 μg/ml) (Mean ± SE of triplicate cultures, one of three experiments). **D**. 3H-Thymidine incorporation at 60 hours of sorted NCD4lo and NCD4hi cells from TCR transgenic OT-II mice to titrating concentrations of OVA-II peptide with irradiated B6 DCs as APCs (mean ± SE of triplicate cultures, one of four experiments). **E**. Amount of IL-2 estimated from supernatants 48 hours post stimulation. Data from one of four experiments. APCs, antigen-presenting cells; DCs, dendritic cells; NCD4, naïve CD4 T cells; SE, standard error; TCR, T cell receptor.

In order to see if these properties of NCD4hi and NCD4lo cells is an intrinsic characteristic in developing NCD4 populations, we tested thymic mature single-positive (SP) CD8-CD4 + CD24-Qa2+ NCD4 cells. When these cells were gated to identify CD4lo and CD4hi cells (Figure 3A, log and linear plots), it was apparent that they show no differences in either cell size (Figure 3A, right panel) or CD5 levels (Figure 3B, log and linear plots). When these CD4lo and CD4hi CD4SP thymocytes were sorted and activated with plate-coated anti-CD3 + anti-CD28 they responded comparably (Figure 3C) unlike NCD4lo and NCD4hi cells from the periphery (Figure 1). These data indicate that the functional and phenotypic differences observed between peripheral NCD4lo and NCD4hi cells are not thymic in origin.

In order to examine if the hyporesponsiveness of NCD4lo cells could be plausibly ascribed to differences in the normal polyclonal TCR repertoire [40], we sorted splenic NCD4hi and NCD4lo cells from mice transgenic for an MHCII-restricted TCR (OT-II) and stimulated these sorted cells with titrating concentrations of the cognate ovalbumin peptide (OVA-II) presented by bone-marrow derived dendritic cells (BMDCs) as antigen-presenting cells (APCs). NCD4lo OT-II cells responded poorly to cognate peptide and produced less IL-2 than NCD4hi OT-II cells did (Figure 3D and E), showing that even in naïve T cells with a single TCR, CD4 levels were correlated with responsiveness.

### Human NCD4lo and NCD4hi cells show properties similar to those from mice

**Figure 4:**
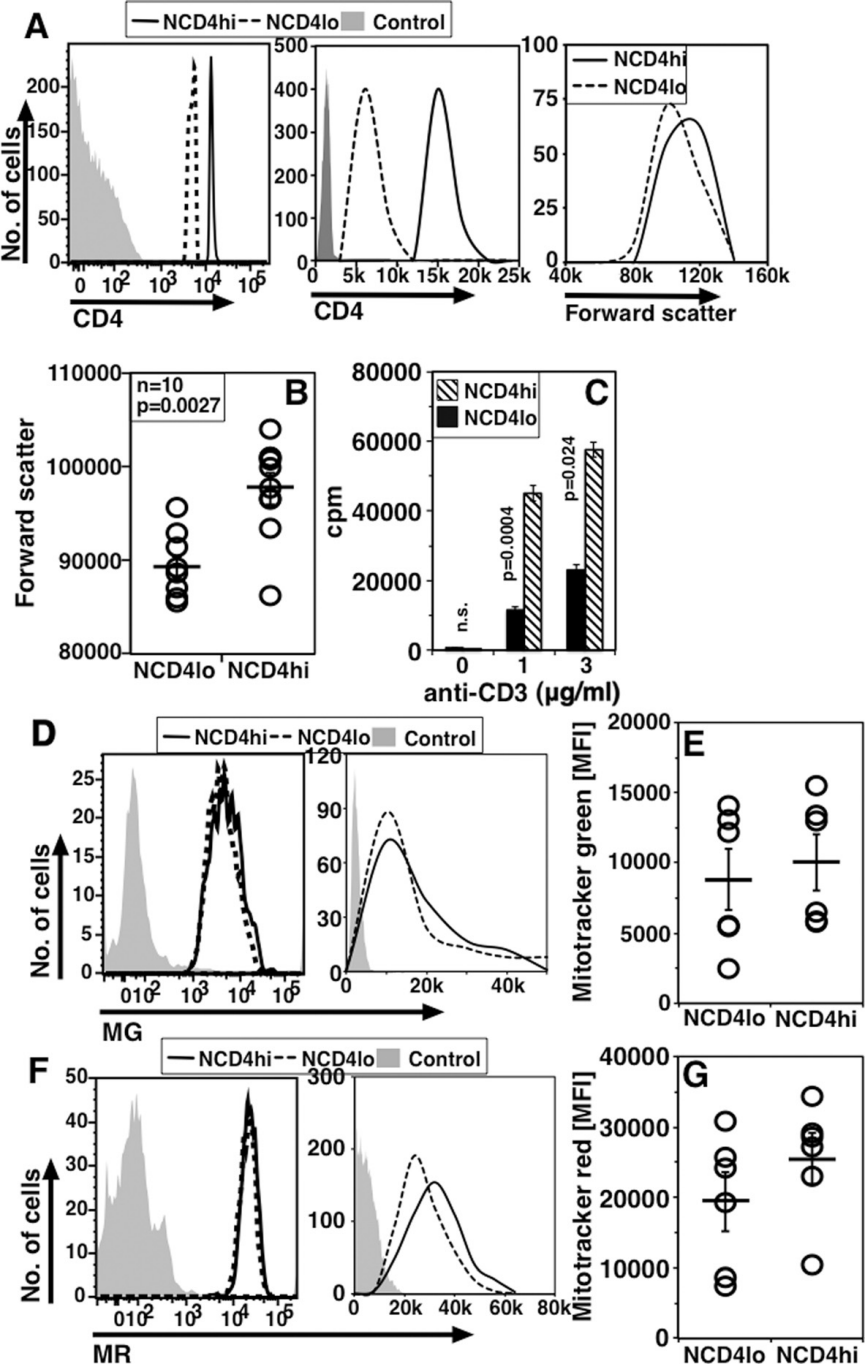
**NCD4lo and NCD4hi cells from human PBMCs show properties similar to mouse cells. A**. A representative gating strategy for NCD4lo and NCD4hi cells (left), histogram overlay of sorted NCD4lo and NCD4hi cells (middle) and analysis of the same cells for size (right). **B**. Forward scatter for NCD4hi and NCD4lo cells from individual donors (Mean ± SE). **C**. 3H-Thymidine incorporation in response to titrating doses of anti-CD3 and 3 μg/ml of anti-CD28 60 hours post-activation (Mean + SE of triplicate cultures; one of four independent donors). **D**. Histogram overlays for MG staining in log (left) and linear (right) scale in NCD4lo and NCD4hi. **E**. MFI for MG on NCD4hi and NCD4lo cells from six individual donors (mean ± SE). **F**. Histogram overlays for MR staining in log (left) and linear (right) scale in NCD4lo and NCD4hi. **G**. MFI for MR on NCD4hi and NCD4lo cells from six individual donors (mean ± SE). MFI, mean fluorescence intensity; MG, Mitotracker Green; MR, Mitotracker Red; NCD4, naïve CD4 T cells; PBMC, peripheral blood mononuclear cells; SE, standard error.

We tested if the distinctions observed between NCD4lo and NCD4hi mouse cells were also found in human CD4 cells. For this, we examined NCD4 cells (CD4+ CD45RA + CD25-) from human peripheral blood mononuclear cells (PBMCs) from healthy donors. When gated NCD4hi and NCD4lo populations (Figure 4A, left panel) were examined, NCD4lo cells were smaller in size in multiple donors (Figure 4A, right panel, Figure 4B), consistent with the mouse data. Sorted human NCD4hi and NCD4lo cells showed about two-fold difference in CD4 mean fluorescence intensity (MFI) values (Figure 4A, middle panel) similar to mouse cells. Upon activation with plate-coated anti-CD3 + anti-CD28 mAbs, NCD4hi cells proliferated much more than NCD4lo cells did (Figure 4C). Mitochondrial mass and membrane potential were also evaluated in these cells using MG and MR (Figure 4D and F respectively; left log plots, right linear plots) as for mouse cells. Similar to mouse NCD4lo cells, human NCD4lo cells, too, showed no striking differences from NCD4hi cells in mitochondrial mass and potential (Figure 4E to G).

### Consequences of peripheral residence on NCD4 T cells

**Figure 5:**
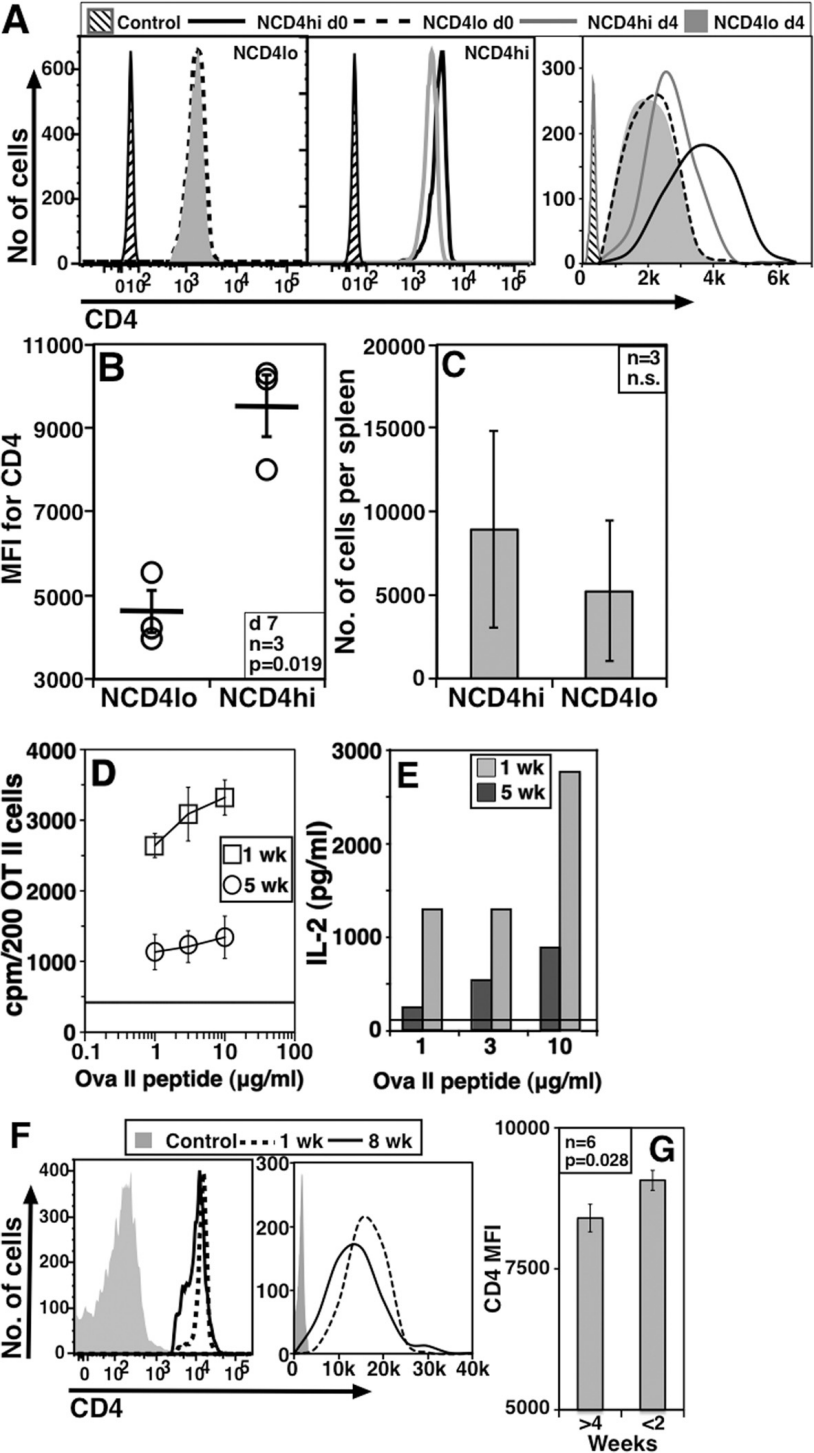
**Consequences of peripheral residence in NCD4 T cells. A**. CD4 levels on sorted, and differentially labeled NCD4lo (left and right) and NCD4hi (middle and right) cells prior to (day 0) and four days after transfer. Left and middle panel – log plots, right panel – an overlay in linear scale (one of three experiments). **B**. CD4 MFI values for NCD4lo and NCD4hi cells seven days post-transfer. Each circle represents an individual recipient mouse and the horizontal line indicates mean ± SE. **C**. Number of transferred NCD4hi and NCD4lo cells recovered from individual recipient mouse spleens seven days post-transfer shown as mean ± SE (one of two experiments). **D**. 3H-Thymidine incorporation in OT-II cells parked in WT mice for one or five weeks in response to titrating concentrations of OVA-II peptide. Data normalized for 200 OT-II cells (Mean ± SE of triplicate cultures; 1 of >7 experiments). Horizontal line indicates background cpm. **E**. Levels of IL-2 in culture supernatants from a representative experiment as in panel D. Horizontal line indicates background IL-2 levels (one of five experiments). **F**. Histogram overlays showing CD4 levels as log and linear plots of one-week-parked or eight-week-parked OT-II cells. **G**. CD4 MFI levels from long-parked (>4 week) and short-parked (<2 week) OT-II cells (Mean ± SE, one of six experiments). MFI, mean fluorescence intensity; NCD4, naïve CD4 T cells; SE, standard error; WT, wild type.

The data so far suggest that the association between CD4 levels and functional phenotype was a peripherally induced characteristic. In order to probe this further, we began by sorting splenic NCD4lo and NCD4hi cells. We labelled these cells with green and violet dyes for identification, mixed them in 1:1 proportion and transferred them intravenously (i.v.) to wild type (WT) recipients. On day 4 post-transfer, donor NCD4 cells were identified in recipient spleens and analyzed for their CD4 levels. Comparison of CD4 levels on NCD4hi and NCD4lo cells before (d0) and after transfer (d4), done in parallel, showed that adoptively transferred NCD4lo cells continued to show nearly unchanged CD4 levels which were lower than transferred NCD4hi cells (Figure 5A, left and middle panels log plots, right panel linear plot). CD4 levels on NCD4hi cells declined from d0 levels to day 4 after transfer, indicating that naïve CD4 T cells lose CD4 levels over time in the periphery. However, despite this decline, NCD4hi cells continued to have higher CD4 levels than NCD4lo cells up to day 7 after transfer (Figure 5B). Notably, there was no appreciable difference in the ability of transferred cells to survive *in vivo*, at least for a period of one week (Figure 5C). These data suggest that CD4 levels do not change randomly *in vivo* but are likely to be reduced over time, perhaps with longer residence.

We also used the lymphoreplete environment of WT congenic mice to analyze the consequences of prolonged peripheral residence on NCD4 T cell function. B6.SJL mice were given 5 × 10^6^ OT-II cells and the proliferative responses of these donor cells *in vitro* were analyzed one to eight weeks later. OT-II cells parked for five weeks *in vivo* responded poorly in terms of proliferation and IL-2 production than those parked for one week (Figure 5D and E). When CD4 levels on these parked OT-II cells were analyzed, OT-II cells parked for one week showed higher CD4 levels compared to cells parked for eight weeks (Figure 5F, log and linear plot). When OT-II cells parked for less than two weeks were compared with those parked for more than four weeks using data from multiple experiments, significant differences were found in their relative CD4 levels (Figure 5G). DO11.10 cells parked in Balb.c mice for six months showed lower CD4 levels compared to those parked for two weeks (see Additional file 3: Figure S3A and S3B).

### Consequences of prolonged tonic signaling and absence of tonic signaling in naïve T cells

**Figure 6:**
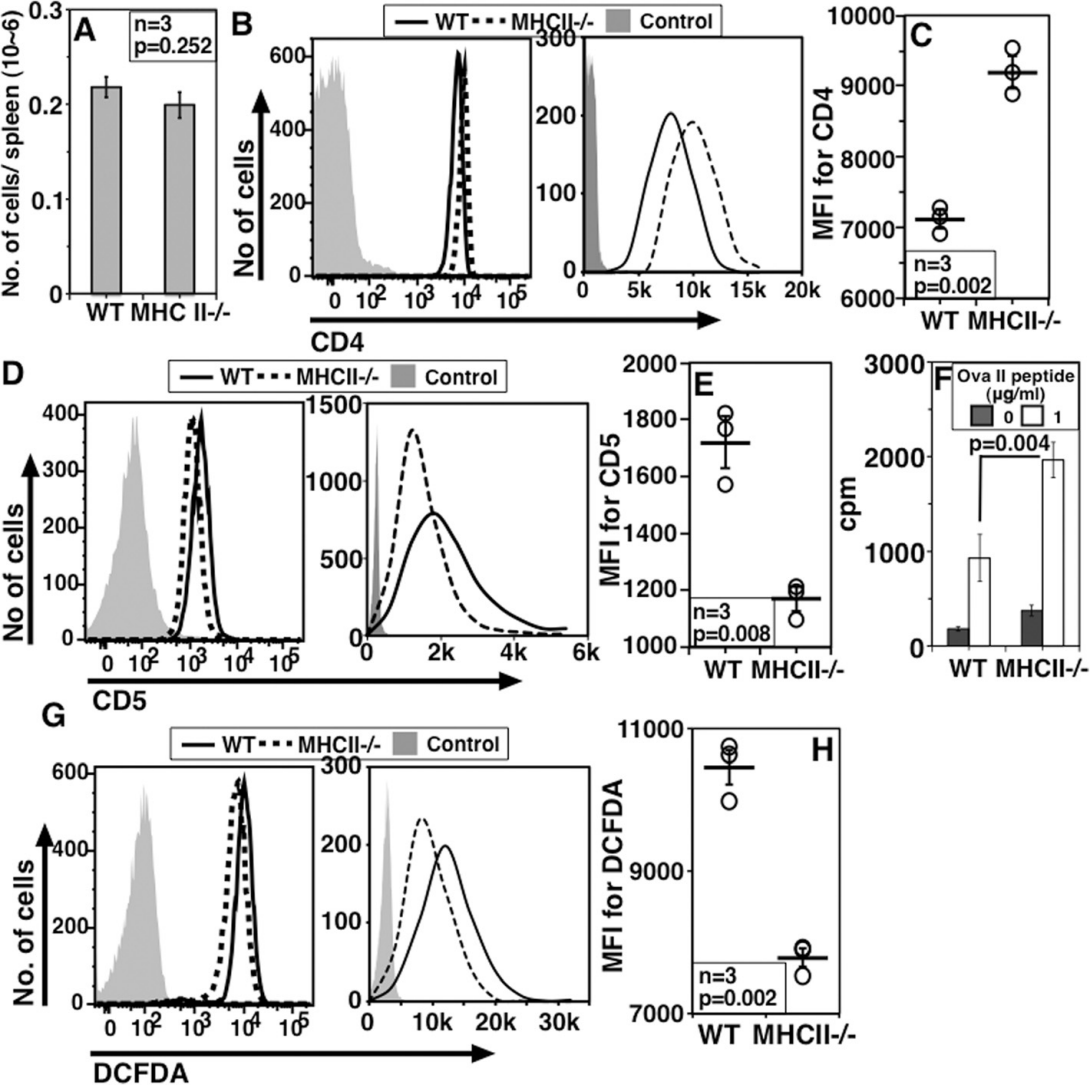
**Consequences of prolonged tonic signaling and absence of tonic signaling in naïve T cells. A**. Numbers of adoptively transferred NCD4 OT-II cells recovered from spleens three days after transfer into WT and MHCII-null mice (one of three independent experiments). **B**. Comparison of CD4 levels (plotted on log on left and linear scale on right) of donor NCD4 OT-II cells after three days in WT and MHCII-null mice. **C**. Pooled data, of paired samples showing CD4 MFI and mean ± SE (one of three experiments). **D**. Comparison of CD5 levels (plotted on log on left and linear scale on right) of donor NCD4 OT-II cells after three days in WT and MHCII-null mice. **E**. Pooled data, of paired samples showing CD5 MFI and mean ± SE (one of three experiments). **F**. Proliferation assay of donor NCD4 OT-II cells from WT or MHCII-null recipient mice four days post-transfer in response to OVA-II peptide in the presence of irradiated DCs from WT mice. Data were normalized for 1,000 donor NCD4 OT-II cells (Mean ± SE of triplicate cultures; data representative of two experiments; three recipient mice per group per experiment). **G**. Representative DCFDA staining (plotted on log on left and linear scale on right) for donor OT-II cells from WT or MHCII-null spleen cells seven days post-transfer. **H**. Pooled data, of paired samples showing DCFDA MFI values (Mean ± SE, one of three experiments) in WT or MHCII-null recipients seven days after transfer. DCs, dendritic cells; DCFDA, 2-7-dichlorofluorescin diacetate; MFI, mean fluorescence intensity; NCD4, naïve CD4 T cells; SE, standard error; WT, wild type.

The data so far suggested the hypothesis that CD4 levels and the responsiveness of NCD4 cells were reduced with increasing duration of peripheral residence. During peripheral residence, MHCII-mediated tonic signals are received by NCD4 cells. To test if the reduction in CD4 levels and the loss of responsiveness were related to MHCII-mediated tonic signaling, we ‘parked’ purified NCD4 OT-II cells by adoptive transfer into either WT or MHCII-null recipient mice. When recipients were euthanized on day 3 post-transfer, the numbers of OT-II cells recovered per spleen from WT and MHCII-null recipients were comparable (Figure 6A) suggesting that there is unlikely to be a major difference in cell survival or homeostatic proliferation in the two groups at this time post-transfer. However, NCD4 OT-II cells recovered from WT recipients had lower CD4 levels (Figure 6B, log and linear plot and 6C) and higher CD5 levels (Figure 6D log and linear plots, and 6E) than cells recovered from MHCII-null recipients. When these cells were stimulated with the cognate OVA-II peptide, OT-II cells parked in MHCII-null recipients responded better than the corresponding cells parked in WT recipients (Figure 6F).

Since accumulation of ROS is a common hallmark of senescent cells [21-23], we examined the possibility that longer parking of OT-II NCD4 cells in WT versus MHCII-null recipients may also lead to differences in ROS levels in them. When NCD4 OT-II cells were parked for ten days in WT or MHCII-null recipients, and their levels of staining intensity for the ROS indicator dye 2-7-dichlorofluorescin diacetate (DCFDA) examined, cells parked in WT mice showed higher ROS levels than cells parked in MHCII-null recipients (Figure 6G log and linear plots, and 6H).

### Prolonged residence *in vivo* leads to loss of functionality and accumulation of ROS in NCD4 cells

**Figure 7:**
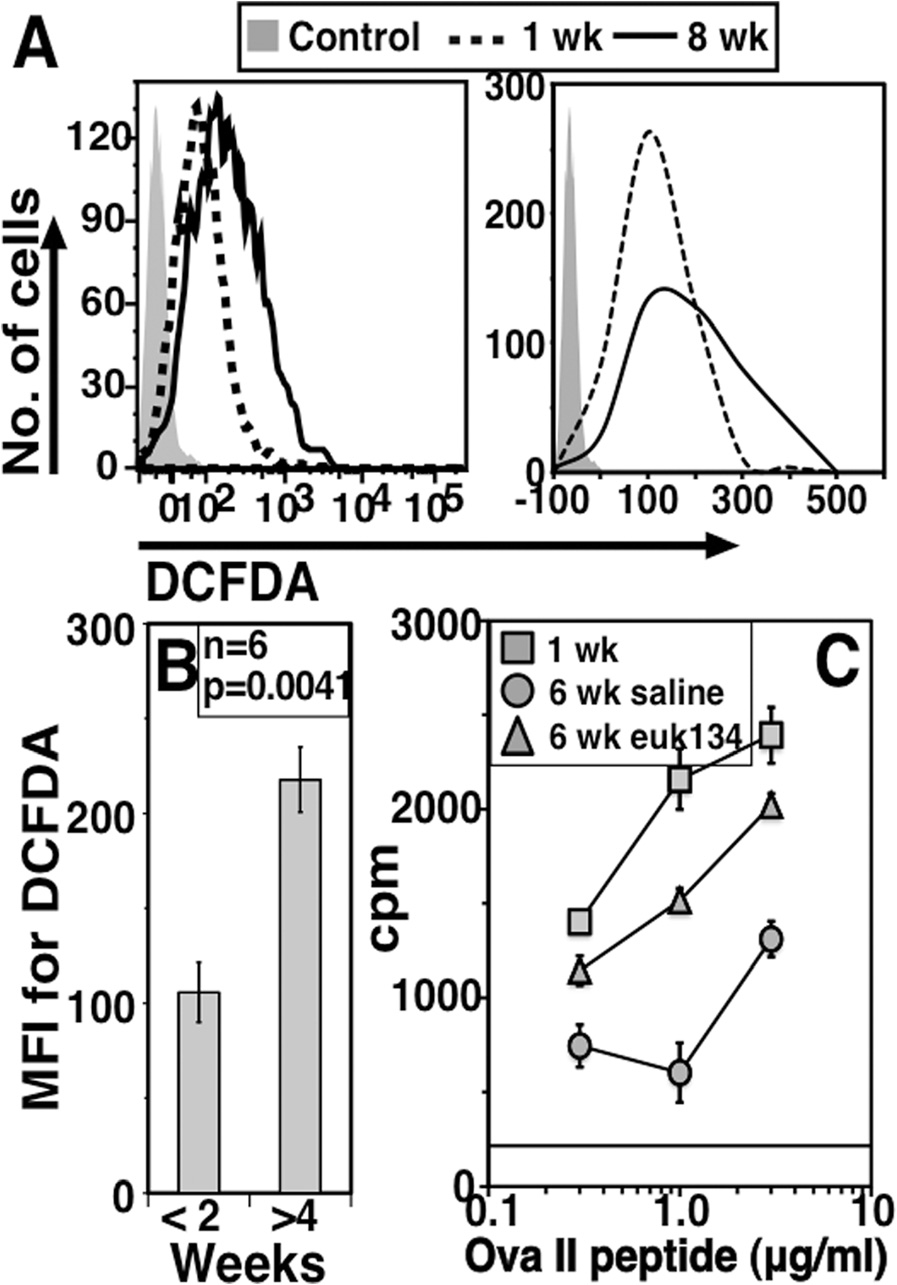
**Prolonged residence** ***in vivo*** **leads to loss of functionality and accumulation of ROS in NCD4 cells. A**. Histogram overlays (plotted on log on left and linear scale on right) for DCFDA from OT-II cells parked for one or eight weeks in WT mice. **B**. DCFDA MFI values from long-parked (>4 weeks) and short-parked (<2 weeks) OT-II cells (mean ± SE, one of three experiments). **C**. 3H-Thymidine incorporation in response to titrating concentrations of OVA-II peptide in OT-II cells parked for one or six weeks in WT mice (with or without Euk-134 treatment). Data normalized to 200 OT-II cells (Mean ± SE of triplicate cultures; one of three experiments). Horizontal line indicates background cpm. DCFDA, 2-7-dichlorofluorescin diacetate; MFI, mean fluorescence intensity; NCD4, naïve CD4 T cells; ROS, reactive oxygen species; SE, standard error; WT, wild type.

We next examined if ROS levels differed in parked cells in normal lymphoreplete mice depending on the duration of parking. OT-II cells parked for eight weeks in WT congenic lymphoreplete mice showed higher intensities of DCFDA staining as compared to cells parked for one week (Figure 7A, log and linear plots). In multiple experiments when OT-II cells parked for less than two weeks were compared with those parked for more than four weeks, significant differences were found in their relative DCFDA staining intensities (Figure 7B).

We next tested if ROS scavenging could ameliorate the relative hyporesponsiveness induced in OT-II cells by prolonged parking. OT-II cells were adoptively transferred into WT recipients that were given alternate-day treatment with either vehicle alone or a superoxide dismutase (SOD)/catalase mimic, Euk-134 [41], for five weeks following adoptive transfer. WT recipients given OT-II cells one week previously were also used as a control group. OT-II cells from Euk-134-treated recipients showed a better proliferative response at the end of six weeks of parking compared to cells from vehicle- treated recipients, although their responses still remained poorer than those of OT-II cells parked for only one week (Figure 7C).

Since ROS level differences had become evident by 10 days or so, we tested if a shorter period of Euk-134 treatment was sufficient to improve reactivity of parked OT-II cells. However, 15-day Euk-134 treatment did not result in any improvement of reactivity (see Additional file 3: Figure S3C). These data suggest that the ROS-mediated contribution to hyporeactivity requires fairly prolonged ROS exposure, and, thus, ROS is clearly not the major factor imposing hyporeactivity on NCD4 cells upon prolonged peripheral residence.

### The Erk-DUSP6-miR-181a axis contributes to hyporesponsiveness of NCD4 T cells during peripheral residence

**Figure 8:**
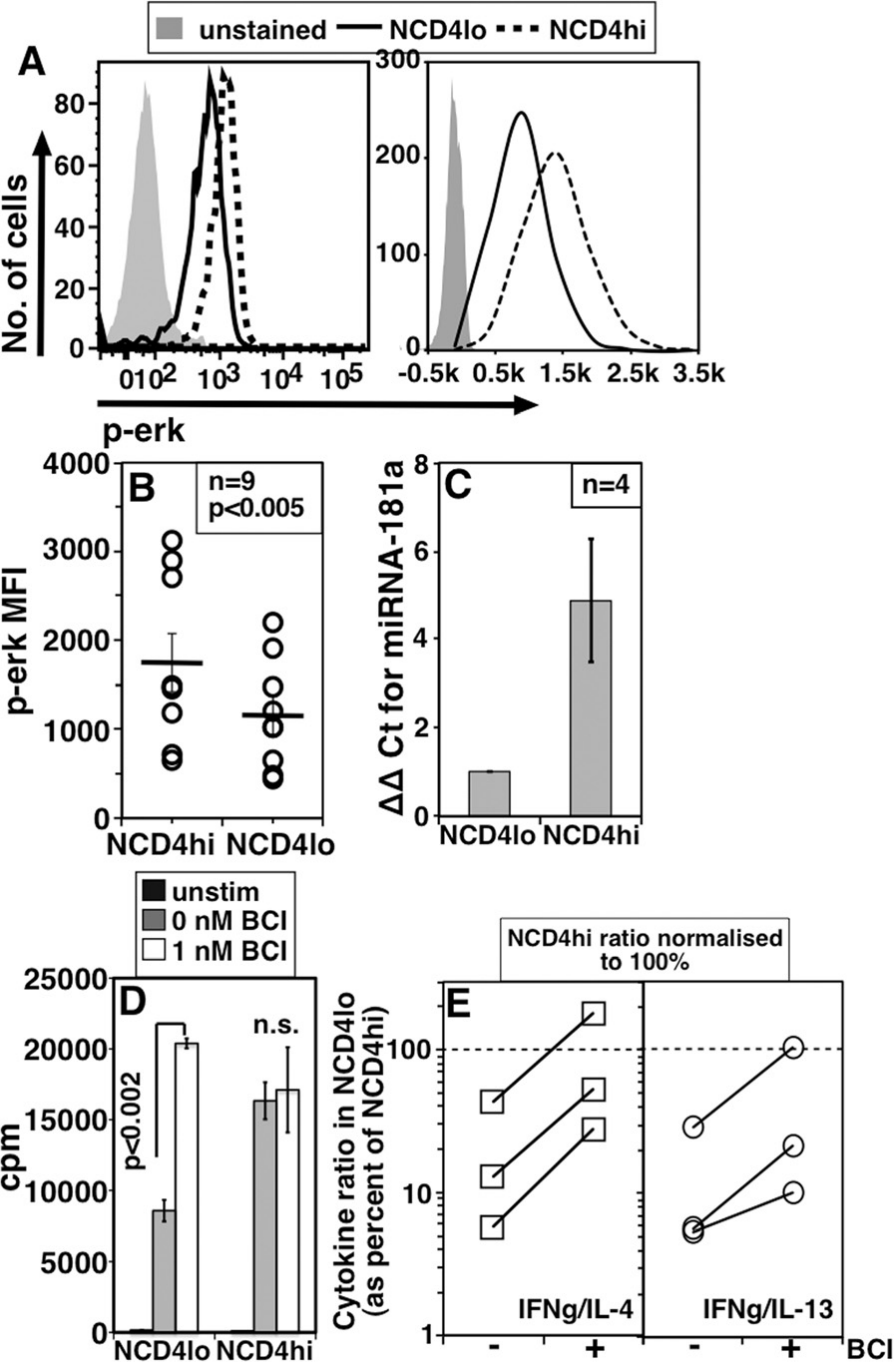
**The Erk-DUSP6-miR181a axis contributes to hyporesponsiveness of NCD4lo T cells. A**. Histogram overlays of baseline *ex vivo* p-Erk (plotted on log on left and linear scale on right) in NCD4lo and NCD4hi cells. **B**. Pooled data of paired samples showing baseline p-Erk MFI values (Mean ± SE) in NCD4lo and NCD4hi cells. **C**. ΔΔCt values of Real time RT-PCR for miR-181a normalized to miR-142 on NCD4hi and NCD4lo cells (Mean ± SE from four sets of independently sorted cells). **D**. 3H-Thymidine incorporation of NCD4lo and NCD4hi cells treated or not with BCI prior to activation (Mean ± SE of triplicate cultures). Data representative of five mice, one of three experiments. **E**. Relative IFNγ/IL-4 and IFNγ/IL-13 ratios for BCI treated or untreated NCD4lo cells during recall response. Data from three independent experiments shown normalized to respective ratios for NCD4hi cells as 100%, shown as a dotted line. BCI, (E)-2-benzylidene-3-(cyclohexylamino)-2,3-dihydro-1H-inden-1-one; DUSP6, dual-specific phosphatase 6; MFI, mean fluorescence intensity; NCD4, naïve CD4 T cells; SE, standard error.

Phosphorylation of mitogen-activated protein (MAP) family tyrosine kinase Erk has been shown to be poor in many aging cells, including T cells [6,8,42,43] suggesting that T cell age may also be correlated with Erk phosphorylation. We examined baseline p-Erk levels *ex vivo* in NCD4hi and NCD4lo cells. NCD4lo cells show significantly lower levels compared to NCD4hi cells (Figure 8A log and linear plot, and 8B). The defective phosphorylation of Erk in human T cells is regulated by the activity of DUSP6, which in turn is controlled by miR-181a [8]. NCD4 cells from older people are reported to express lower levels of miR-181a (relative to a control miRNA, miR-142) than NCD4 cells from young people [8]. We therefore estimated the levels of miR-181a and miR-142 transcripts in mouse T cells using real-time RT-PCR assays. Levels of miR-181a transcripts (normalized to miR-142 transcript levels) were lower in NCD4lo cells compared to NCD4hi cells (Figure 8C), further suggesting that because of longer peripheral residence NCD4lo cells show a pattern similar to that reported in NCD4 T cells from older individuals [8]. Consistent with the role of miRNA-181a in suppressing DUSP6 expression [8], when NCD4lo and NCD4hi cells were treated with a pharmacological inhibitor of DUSP, (E)-2-benzylidene-3-(cyclohexylamino)-2,3-dihydro-1H-inden-1-one (BCI) prior to activation, NCD4lo cells responded better following treatment with BCI (Figure 8D), while NCD4hi cells showed no effect of BCI. Further, analysis of cytokines secreted by NCD4lo and NCD4hi cells treated with BCI and differentiated *in vitro* showed that the relative Th2 tendency of NCD4lo cells was partially reversed by BCI treatment (Figure 8E).

### Phenotypic and functional features of NCD4 cells from young mice with longer peripheral residence are similar to NCD4 cells from aged mice

**Figure 9:**
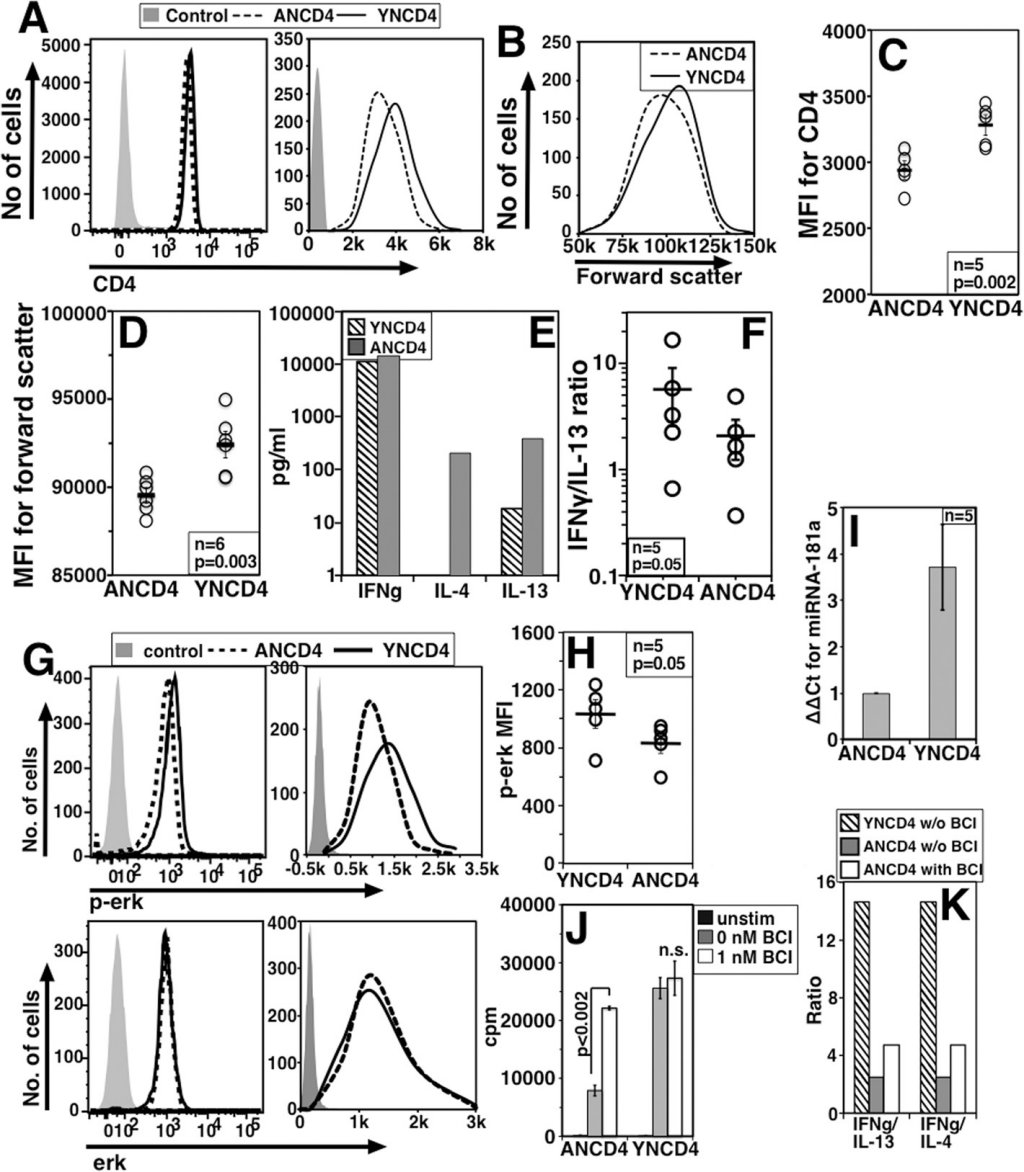
**NCD4lo cells resemble naïve CD4 T cells from aged mice in their phenotypic and functional attributes. A**. CD4 levels on ANCD4 and YNCD4 cells (plotted on log on left and linear scale on right) stained as a mixture in a single well. **B**. Representative forward scatter histograms for ANCD4 and YNCD4 cells. **C**. Comparison of CD4 MFI values of ANCD4 and YNCD4 cells (Mean ± SE; data representative of three experiments). **D**. Data for forward scatter from individual mice (Mean ± SE; data representative of three experiments). **E**. Representative data showing levels of cytokines in supernatants of *in vitro* primed and recalled YNCD4 and ANCD4 cells. **F**. IFNγ/IL-13 ratios and mean ± SE calculated based on absorbance values. **G**. Histogram overlays of baseline *ex vivo* p-Erk (top) and total Erk (bottom) in ANCD4 and YNCD4 cells (plotted on log on left and linear scale on right). **H**. Pooled data, of paired samples showing baseline p-Erk MFI values (Mean ± SE) in ANCD4 and YNCD4 cells. **I**. ΔΔCt values of Real time RT-PCR for miR-181a normalized to miR-142 on ANCD4 and YNCD4 cells (Mean ± SE from five sets of independently sorted cells). **J**. 3H-Thymidine incorporation of ANCD4 and YNCD4 cells treated or not with BCI prior to activation (Mean ± SE of triplicate cultures). Data representative of six mice, one of three experiments. **K**. IFNγ/IL-13 and IFNγ/IL-4 ratio for YNCD4, BCI treated ANCD4 or BCI untreated ANCD4 cells during recall response. Data representing one of four independent experiments. ANCD4, naïve CD4 T cells from aged mice; BCI, (E)-2-benzylidene-3-(cyclohexylamino)-2,3-dihydro-1H-inden-1-one; MFI, mean fluorescence intensity; SE, standard error; YNCD4, naïve CD4 T cells from young mice.

Average peripheral residence time of naive T cells in aged mice and older people is reported to be longer than their younger counterparts due to decreasing thymic output with age [16,17]. Since our data above suggest that NCD4lo cells from young mice are likely to be those with longer peripheral residence, we compared NCD4 cells from young (YNCD4) and aged (ANCD4) mice for functional and phenotypic features. We have already shown that ANCD4 cells respond poorly and die more easily post-activation [34]. We observed that ANCD4 cells had modestly but consistently lower CD4 levels than YNCD4 cells (Figure 9A log and linear plot, and 9C). ANCD4 cells were also consistently smaller than YNCD4 cells (Figure 9B and D). We also looked at the ability of sorted ANCD4 and YNCD4 cells to differentiate *in vitro* in response to non-polarizing activating conditions. Supernatants from cultures of ANCD4 and YNCD4 cells primed *in vitro* with anti-CD3 + anti-CD28 for three days and then restimulated with titrating doses of anti-CD3 were analyzed for IFNγ, IL-4 and IL-13. ANCD4 cells thus differentiated to effector cells produced more IL-4 and IL-13 compared to YNCD4 cells (Figure 9E). The relative Th1 dominance, indicated by the IFNγ/IL-13 ratio, was significantly lower in differentiated ANCD4 cells (Figure 9F). We also examined total and p-Erk levels. While total Erk levels (Figure 9G log and linear scale, bottom plot) were not different between ANCD4 and YNCD4 cells *ex vivo*, p-Erk levels in the resting state were lower in ANCD4 cells (Figure 9G log and linear scale, top plot). Differences in the pErk levels were significant (Figure 9H). In multiple independent experiments, relative levels of miR-181a were lower in ANCD4 cells compared to YNCD4 cells (Figure 9I) extending observations reported on human cells [8]. Consistent with the role of miRNA-181a in suppressing DUSP6 expression [8], ANCD4 cells responded better when treated with a pharmacological inhibitor of DUSP, BCI, prior to activation (Figure 9J). It is noteworthy that there was no apparent effect of BCI on YNCD4 cells, despite the fact that some of the YNCD4 cells would be expected to be CD4lo and would, therefore, have shown an effect of BCI. It is likely that the contribution of NCD4lo cells in the bulk YNCD4 cells may not be statistically apparent. Further, analysis of cytokines secreted by ANCD4 and YNCD4 cells treated with BCI and differentiated *in vitro* showed that the relative Th2 tendency of ANCD4 cells was partially reversed by BCI treatment (Figure 9K).

## Discussion

Functional variations in apparently homogeneous cell populations have been recognized based on gene induction [27] as well as expression of protein levels [28,29]. The origins of such variation can be either stochastic and/or can lie in the different experiential histories of individual cells. Our data here show that differences in CD4 levels in an NCD4 cell population showing unimodal distribution are associated with differences in cell size, CD5 expression levels, and ability to respond, differentiate and die post-activation. Our data also indicate that these differences are not purely stochastic but are influenced by microenvironmental MHCII-mediated signals received by peripheral NCD4 cells. Finally, we also show that the resultant variations in responsiveness are influenced by ROS accumulation and DUSP function, providing entry points for envisaging immunotherapeutic interventions in older people.

Despite the unimodal distribution, the gated top and bottom CD4 level deciles of NCD4 cells show distinct differences in both mice and humans. The NCD4lo cells respond poorly to activation, and their response matures in a more Th2-skewed direction. CD4 is a co-receptor and signals mediated by CD4-MHCII interaction can contribute to peptide-MHCII-mediated signaling for activation in naive T cells [44]. However, the anti-CD3 + anti-CD28 activation we have used would be expected to bypass any direct contribution of CD4. The CD4 molecules on responding NCD4 cells are antibody-engaged in our purification protocols, and there is some evidence for TCR-independent signaling capabilities of CD4 molecules [44]. However, our data on proximal signaling events such as Zap-70 or Lck phosphorylation and calcium flux induction show no evidence for their modification during activation of NCD4lo and NCD4hi cells, indicating that differential CD4-mediated signals induced by the anti-CD4 antibody are not responsible for the differences we observe, and that the differences in CD4 levels *per se* are unlikely to be directly involved in the hyporesponsiveness of NCD4lo cells. It is more likely that CD4 levels are simply correlates of global alterations in these populations. Notably, NCD4lo and NCD4hi cells differ in their IL-2 production, a characteristic property of ‘anergic’ CD4 T cells [45]. However, exogenous IL-2 supplementation only partially corrects the proliferative deficit in NCD4lo cells, and NCD4lo cells remain quite capable of generating CD4 T cell ‘effectors’ on activation, albeit with an altered cytokine balance, indicating that simple anergy is unlikely to be the major modification involved.

Our data not only show that the unimodal distribution contains functionally distinct subsets but that they differ phenotypically as well. The NCD4lo cells have lower CD2 and MHCI levels, but higher CD5 and CD54 levels. They are smaller, too, which may indicate some metabolic downmodulation, although mitochondrial mass and transmembrane potential are not notably altered in them, suggesting that mitochondrially mediated cell size determining factors may not be causal [46,47]. Non-mitochondrial possibilities, such as some potassium channels that are reported to regulate homeostatic volume in T cells, would need to be examined [48].

Notably, none of the functional and phenotypic differences between peripheral NCD4lo and NCD4hi T cells are visible in thymic CD4SP cell subsets, suggesting that the differences arise peripherally rather than being programmed during thymic development. There are interesting implications of the observation that even the mature CD24- thymic CD4SP cells do not show functional distinctions between their CD4lo and CD4hi sub-populations. This indicates that, in thymic CD4SP cells, variation in CD4 levels is indeed likely to be merely stochastic and without functional consequences, as would be expected if the normal distribution of CD4 levels in a cell population was due to biological noise and not any functional heterogeneity. These findings lend interpretative weight to our data with peripheral NCD4 cells which do show that their CD4 levels correlate with functional heterogeneity and time of peripheral residence.

It may be noted that any more direct functional comparisons between CD4SP thymocytes and peripheral NCD4hi cells are difficult to make, since it is quite likely that NCD4 reactivity is low in mature thymocytes for reasons unrelated to cellular age, because recent thymic emigrants in the periphery are poorly responsive and take some time to reach peak reactivity [49,50].

One obvious distinction between lymphocyte subpopulations can be envisaged based on clonal repertoire diversity. However, relatively monoclonal TCR-transgenic NCD4 cells also show similar differences. Thus, clonal repertoire diversity is not an absolute prerequisite for these differences.

CD5 levels on T cells have been linked to adaptation and fine-tuning of T cell responsiveness [12,51], possibly via tonic signaling through the restricting MHC element which has been shown to be required for naïve T cell survival, maintenance and homeostatic proliferation in lymphopenic environments [52]. The correlation between CD5 levels on naive T cells and their responsiveness is somewhat controversial. Recently, higher CD5 levels have been correlated with better responsiveness of NCD4 [1]. On the other hand, CD5 has been shown to be a negative regulator of TCR-mediated activation [53,54]. There are also other reports, consistent with our findings, that tonic spMHC contact for both CD4 and CD8 T cells leads to both upregulation of CD5 levels and to induction of hyporeactivity [12,13]. The differences in the correlation of CD5 levels on T cells with higher or lower reactivity may be related to the origin of the differences in CD5 levels; thus, it is possible that between naive T cells that have received tonic signals for comparable durations, higher CD5 levels may identify better-responding T cells as reported [1]. However, in some situations, CD5 levels may predominantly reflect the duration of tonic signaling as noted in previous literature [12,13] and in our data here, and in that context, CD5 levels may be inversely correlated with reactivity.

Notwithstanding these issues, it is currently plausible to hypothesize that NCD4lo cells, which have higher levels of CD5, may have received much more tonic signal, possibly simply by having spent more time in the periphery. Consistent with this possibility, when NCD4hi cells are purified and transferred *in vivo*, their CD4 levels slowly go down. This does not happen to NCD4lo cells, possibly indicating that they have already gone as low as possible in their CD4 levels. Further, such parked cells steadily become hyporesponsive over time, again implicating time of peripheral residence as a regulatory influence. Thus, within a unimodal population of NCD4 cells, it is likely that there are multiple cohorts of cells with varying peripheral residence times and, as a result, varying functionality.

An obvious possibility regulating this global alteration over residence time *in vivo* is MHC-mediated tonic signaling which, while it is necessary for the survival of naive T cells, has also been reported to be responsible for attenuation of the response potential of these cells, possibly through the negative regulatory function of CD5 [12,13]. Indeed, parking NCD4 T cells in a MHCII-deficient environment with an acute lack of tonic signaling, for even a short three-day period of parking, reveals MHCII-dependent differences in CD4 levels, CD5 levels and in responsiveness. However, cell size does not change over this time window, indicating that it is not necessary for the hyporesponsiveness.

We observe that ROS levels go up in NCD4 cells as time of peripheral residence increases, whether in the severe situation of parking in MHCII-deficient mice, or in the more physiological situation of parking in WT lymphoreplete mice. In T cells, the mammalian Ste20-like protein kinase Mst-1 is known to regulate SOD-2 and catalase levels [55], and Mst-1-deficient T cells, in addition to showing lower levels of SOD2 and catalase, have higher ROS levels and are sensitive to apoptosis. The synthetic mimetic of catalase and SOD used here, Euk134, has been used as an anti-oxidant *in vivo* for a variety of outcomes ranging from prevention of heart failure post-hypertension [41] to provision of pain relief [56] in situations where ROS accumulation has been implicated in the pathology.

In NCD4 cells, ROS does contribute to the functional phenotype, since ROS scavenging significantly (though not completely) protects NCD4 cells from prolonged six-week parking-induced hyporesponsiveness. However, this ROS-mediated contribution to hyporesponsiveness requires fairly prolonged ROS exposure since 15-day anti-oxidant treatment does not improve reactivity. Thus, while ROS does make a contribution at late stages, it is clearly not the major factor imposing hyporeactivity on NCD4 cells upon prolonged peripheral residence.

If tonic signaling eventually leads to hyporesponsiveness, indications of such a change may be detectable as altered baseline signaling activity [57,58]. In keeping with this, baseline p-Erk levels are lower in NCD4lo T cells. Since Erk phosphorylation has been reported to be controlled by DUSP6 in human T cells, where DUSP6 is regulated by the miR-181a, it was plausible to examine the miR-181a-DUSP axis for a role in the functional differences between NCD4lo and NCD4hi cells. Indeed, miR-181a levels are lower in NCD4lo cells, and a DUSP inhibitor rescues both the poor responsiveness and the Th2-skewing of these cells. Thus, the miR-181a-DUSP axis is at least one mechanism contributing to the alteration of functionality over peripheral residence time in NCD4 cells.

It is noteworthy that NCD4 T cells from aged animals also show hyporesponsiveness similar to that of NCD4lo cells from young mice that we observed. As thymic output decreases with age, naïve T cells in the periphery appear to be longer-lived [16-18,31], and the average peripheral residence time of naïve T cells is greater in aged than in young individuals [32,33]. As would be expected from this, the functional and phenotypic characteristics related to peripheral residence time that our data identified, low CD4 levels, small cell size, poor responses and Th2-skewing, were more prominent in NCD4 T cells from aged mice. While the CD4 T cell defect in aged animals is likely to be multi-factorial in origin [59,60], our data indicate that increased average peripheral residence time of the cells is likely to be one contributory factor.

Finally, our data also show that another molecular component regulating the hyporesponsiveness shared by both ANCD4 and NCD4lo cells is the Erk-DUSP6-miR-181a axis. While TCR-mediated activation of T cells from older humans and mice show many early-stage defects in synapse formation and in phosphorylation-mediated activation of critical kinases [52,61,62], recent data suggest that increased DUSP6 activity in naïve CD4 T cells from older humans may be important in reducing their ability to respond to TCR-mediated stimulation [8]. Our data show that miR-181a levels are lower in both ANCD4 cells and in NCD4lo cells, and the DUSP inhibitor enhances proliferative responses of both ANCD4 and NCD4lo cells. It is interesting to note that, although YNCD4 cells would consist of NCD4hi, NCD4lo and NCD4-intermediate cells, the DUSP inhibitor does not show any significant improvement in proliferative responses of bulk YNCD4 cells. It is plausible that the contribution of NCD4lo cells in the bulk YNCD4 cells may not be statistically apparent in the assays we have used. Our findings extend the reported findings from the human system to the mouse system, and establish further concordance between ANCD4 and NCD4lo T cells and confirm that this axis may indeed be specifically affected during cellular aging. While linkages between increased ROS levels and phosphatase activity of DUSP6 have been shown in non-T cells [63,64] whether such a link exists in aging NCD4 T cells is not yet clear.

## Conclusions

Our data show that, despite unimodal distribution of CD4 on NCD4 cells, there are subsets of NCD4 cells that differ in their peripheral residence time. During this period, they are in receipt of MHCII-mediated signals and show alteration of phenotype and functionality via ROS and DUSP activity. Our findings thus indicate the feasibility of potential pharmacological interventions via small molecules serving either as anti-oxidants or as DUSP inhibitors, directed specifically at CD4 T cells during vaccination of older people.

## Methods

### Mice

All mice were obtained from Jackson Laboratories (Bar Harbor, ME, USA) and bred at the Small Animal Facility of the National Institute of Immunology, New Delhi, India. The following mouse strains were used: C57BL/6 (B6; H-2b, CD45.2, CD90.2), B6.SJL (H-2b, CD45.1), MHCII−/− (H-2b), Balb.c (H-2d) and TCR-transgenic (Tg) OT-II (H-2b), and DO11.10 (H-2d). At the initiation of experiments all mice were six- to eight-weeks of age, except aged B6 mice which were >18 months of age. OT-II mice were crossed with B6.SJL mice, F1 progeny were screened for the presence of the transgene to identify TCR-Tg mice which were further used, as appropriate, for each experiment. Cells from DO11.10, OT-II or OT-IIxB6.SJL mice were adoptively transferred i.v. to appropriate recipients.

### Adoptive transfer experiments

Mouse spleen cells (containing 5 × 10^6^ OT-II or DO11.10 cells per mouse) or further sorted populations, as mentioned wherever appropriate, suspended in normal saline, were transferred into anesthetized recipient mice i.v. Cells were parked in the recipient mice for various time periods as mentioned. For transfer to B6 and MHCII−/− mice, NCD4 cells were sorted from OT-IIxB6.SJL mice and 5 × 10^6^ cells were transferred i.v. Three or ten days post-transfer recipient mice were euthanized and transferred NCD4 cells were distinguished from endogenous NCD4 cells using CD45.1 as a marker. Euk134 was given intraperitoneally (i.p.) (25 mg/kg) on alternate days for two or five weeks [41] with dimethysulfoxide in normal saline as control.

### Reagents

For all cell culture experiments, Roswell Park Memorial Institute (RPMI)-1640 (Biological Industries, Beit Haemek, Israel) with 10% heat inactivated fetal bovine serum (Euroclone, Pero, Italy), 2 mM glutamine, 55 nM β-mercaptoethanol (Sigma-Aldrich, Bengaluru, India), antibiotics and antimycotics (Sigma-Aldrich) was used. Mouse CD2, CD4, CD5, CD8, CD24, CD25, CD44, CD54, CD62L, CD69, Qa2, KJ1.26, TCRβ, MHC-I, Zap-70, pZap-70, Lck, p-Lck, Erk, p-Erk and human CD4, CD45RA, CD25 were detected with specific fluorochrome-coupled anti-mouse or anti-human Abs (BD Biosciences, New Jersey, USA; eBioscience, San Diego, CA, USA; BioLegend, San Diego, CA, USA; Cell Signaling Technology, Danvers, MA, USA; Millipore, Merck, Darmstadt, Germany). Fluo-3 AM and Fura-Red AM (Life Technologies, Carlsbad, CA, USA) were used to study calcium flux. For scoring mitochondrial mass and potential, Mitotracker Green (MG) and Mitotracker Red (MR) (Molecular Probes, Carlsbad, CA, USA) were used respectively. For estimation of ROS, cells were incubated with the cell-permeant dye 2-7-dichlorofluorescin diacetate (DCFDA, Molecular Probes). For anti-oxidant treatment, Euk-134 (Cayman Chemical, Ann Arbor, Michigan, USA) was dissolved in dimethysulfoxide (Sigma-Aldrich) and diluted to the required concentration in normal saline. For inhibiting DUSP in T cells (E)-2-benzylidene-3-(cyclohexylamino)-2, 3-dihydro-1H-inden-1-one (BCI, Sigma Aldrich) was used.

### Isolation and purification of cells

Following euthanasia, single cell suspensions of mouse spleen cells were prepared, subjected to red cell lysis, and stained with anti-mouse CD4, CD25, CD44 and CD62L Abs and electronically sorted (FACSAria III, BD) for naïve CD4 T cells (CD4 + CD25-CD44-CD62L+). For sorting NCD4lo and NCD4hi cells, further gating was done for the approximately 10% NCD4lo and approximately 10% NCD4hi populations in the NCD4 gated population. For sorting CD4lo and CD4hi mature single positive thymic cells, thymic single cell suspensions were made from young B6 mice, stained with CD4, CD8, CD24 and Qa2. CD4 + CD8-CD24-Qa2+ cells identified as mature CD4 cells were further gated for CD4lo and CD4hi and sorted as for peripheral cells.

Blood from healthy, young, consenting adults between the ages of 22 and 35 years (n = 10) with equal representation of men and women was collected and PBMCs separated from heparinized blood by density gradient centrifugation using Ficoll-PaqueTM PREMIUM (GE Healthcare Biosciences AB, Little Chalfont, UK). For sorting human NCD4hi and NCD4lo cells, CD4 + CD25-CD45RA+ low forward scatter cells were gated for NCD4lo and NCD4hi as described for mouse cells and sorted. After sorting, individual fractions were >98% pure in all cases.

### Staining of cells for phenotypic analysis by flowcytometry

Splenic cells from a six- to eight-week old B6 or B6.SJL mouse spleen were identified as NCD4 cells (CD4 + CD44-CD62L + CD25-), highest and lowest deciles were identified on CD4 histogram as NCD4hi and NCD4lo and analyses of their MFI values for CD2, CD4, CD5, CD54, MHC-I, TCRβ, forward scatter, p-Erk, pZap-70, MG and MR were done as paired samples. Splenic cells from 18-month old B6 mice were mixed in equal numbers with splenic cells from six- to eight-week old B6.SJL mice and stained for CD45.1, CD45.2, CD4, CD44, CD62L and CD25 to identify naïve CD4 cells. MFI values on these cells for various markers were compared as paired samples in a given assay for statistical analysis.

For evaluation of proximal signaling, splenic cells (approximately 1 × 10^7^) were loaded with 0.5 μM of Fluo-3 AM and 1.5 μM of Fura-Red AM at 37°C, followed by surface staining for identifying NCD4 cells at 4°C. Stained cells were brought back to 37°C and baseline readings obtained. T cells were activated with anti-CD3 + anti-CD28 (10 + 3 μg/ml, respectively), followed by ionomycin (Sigma). For pZap-70 and pLck a similar staining and activation protocol was followed without using dyes mentioned for calcium measurements.

For evaluating the stability of NCD4hi and NCD4lo cells *in vivo*, cells were sorted from six- to eight-week old B6 mice. NCD4hi cells were labelled with CFSE whereas NCD4lo cells were labelled with CellTrace Violet. A total of 2 × 10^6^ NCD4hi cells were mixed with 2 × 10^6^ NCD4lo cells and transferred to age-matched B6 recipients. An aliquot of a mixture of cells was cryopreserved in 10% dimethyl sulfoxide (DMSO) containing FCS at −70°C. Mice were euthanized four or seven days later and their splenic cells were similarly cryopreserved and thawed along with cells cryopreserved on d0. These cells were stained for CD4, CD44 and CD62L and transferred cells in donor splenocytes were identified based on the dyes used for staining before transfer. Relative CD4 expression levels on these populations on the day of transfer were compared with those on day 4 post-transfer. Spleens from recipient WT and MHCII−/− mice were stained in parallel to obtain MFI values for CD4, CD5 and DCFDA on transferred cells and treated as paired samples for statistical analysis.

For long-term parking experiments involving parking period up to eight weeks, OT-II (CD45.2) cells were transferred to B6.SJL mice and parked for differing periods as indicated. Recipient mice were euthanized on the same day and MFIs of CD4 and DCFDA on OT-II cells with different parking periods were compared as paired samples for statistical analysis. DO11.10 cells transferred in Balb.c mice were identified as CD4 + KJ1.26+ cells. Recipient mice with six months or two weeks parking times were euthanized on the same day and CD4 levels compared.

For phenotypic analysis of human PBMCs, NCD4 cells were identified as CD4 + CD45RA + CD25-. NCD4hi and NCD4lo subsets were further identified as described for mouse cells. PBMCs were stained for MG and MR. MFIs for CD4, forward scatter, MG and MR were compared on NCD4hi and NCD4lo cells as paired samples for statistical analysis.

For all the stainings, protocols recommended by the manufacturers were used.

### *In vitro* assays

All sorted cells were incubated in FCS-containing medium at 37°C for two to three hours before setting up functional assays. For naïve T cell stimulation, plates were coated with a mixture of functional grade anti-mouse CD3 and anti-mouse CD28 Abs (e-Biosciences) or purified anti-human CD3 and anti-human CD28 Abs (BD Biosciences). Optimum concentrations of mAbs were determined prior to use. Human CD4 cell cultures were supplemented with 30 U/ml recombinant IL-2 (Roche, Basel, Switzerland). For OT-II cell stimulation, titrating doses of OVA-II peptide (a.a. 323–339, Peptron Inc., Daejeon, Republic of Korea) were used along with BMDCs as APCs obtained as described earlier [65]. Proliferation was measured by 3H-thymidine (Perkin Elmer, Waltham, MA, USA) incorporation in the last 8 to 16 hours of a 60 to 72 hour assay. CD69 upregulation was measured by flow cytometry 16 to 20 hours post-activation. Proliferation of polyclonal NCD4hi and NCD4lo cells by anti-CD3 + anti-CD28 mAbs was also measured by CFSE dilution, where at the end of 60 to 72 hours dead cells staining positive with Sytox Red were gated out before comparing proliferation curves.

For polyclonal naïve T cell differentiation, purified mouse cells were activated as above for 72 hours, rested in IL-2 (5 U/ml) for 24 hours and restimulated on plates coated with anti-CD3 + anti-CD28 for 24 hours to collect supernatants. Amounts of secreted IL-2, interferon-gamma (IFNγ), IL-4 and IL-13 were analyzed using commercial reagents (eBioscience) and following recommended protocols. Concentrations were calculated from absorbance values using recombinant standards.

In all flow cytometric measurements, dead cells were identified by Sytox red (Molecular Probes) or Annexin-V (BD Biosciences) staining and excluded. In death assays *in vitro*, dead cells in cultures of sorted cell populations were identified by microscopy as trypan blue-stained cells. For inhibition of DUSP6, cells were treated with BCI (1 nM) for one hour at 37°C and washed prior to activation. The optimum, non-toxic concentration of BCI was determined for mouse cells prior to use.

### Real time PCR for miR-181a and miR142

RNA was extracted from approximately two million purified NCD4hi, NCD4lo, ANCD4 and YNCD4 cells using Trizol (Invitrogen, Carlsbad, CA, USA) following the manufacturer’s instructions. For cDNA synthesis 1 μg RNA was used for each sample using the MiScript HiSpec buffer from the miScript-II RT kit to get mature miRNA (Qiagen, Limburg, Netherlands). The miRNA real time PCR assays were performed for mature miRNAs using commercial kits (miScript Primer Assays for Mm_miR-181a_2, targeting the sequence AACAUUCAACGCUGUCGGUGAGU, along with miScript SYBR Green PCR kit, both from Qiagen). Recommended protocols were used to obtain Ct values. ΔΔCt values were calculated using miRNA-142 as a normalizing control (miScript Primer Assays for Mm_miR-142-5p_1 targeting CAUAAAGUAGAAAGCACUACU, from Qiagen) based on a published report [8].

### Data analysis

Flow cytometric data were analyzed using FlowJo (Treestar, San Carlos, CA, USA). Microsoft Excel was used for linear histogram plotting of flow cytometric data. For statistical analysis on paired samples, either Student’s paired ‘t’ test or non-parametric tests, such as the Wilcoxon rank sum test, was used. Student’s ‘t’ test or the Mann–Whitney u test were used for other samples. Values of *P* <0.05 were considered statistically significant.

### Study approval

All mice were maintained and used according to the guidelines of, and with the approval of, the Institutional Animal Ethics Committee of National Institute of Immunology. Collection of human healthy volunteer blood was done by venipuncture after obtaining written informed consent, with due prior approval of the Institutional Human Ethics Committee of National Institute of Immunology. All the experimental work was done at the National Institute of Immunology from where ethical permissions were obtained.

## Additional files

Additional file 1: Figure S1. Additional figure providing NCD4lo and NCD4hi functional features. Additional file 2: Figure S2. Additional figure providing NCD4lo and NCD4hi phenotypic and functional features. Additional file 3: Figure S3. Additional figure providing data on NCD4 levels in an independent TCR-Tg mouse strain and shorter treatment of Euk-134 *in vivo*.

## Abbreviations

ANCD4: naïve CD4 T cells from aged mice
APCs: antigen presenting cells
BCI: (E)-2-benzylidene-3-(cyclohexylamino)-2,3-dihydro-1H-inden-1-one
CFSE: carboxyfluorescein succinimidyl ester
DCFDA: 2-7-dichlorofluorescin diacetate
DCs: dendritic cells
DUSP6: dual-specific phosphatase-6
FCS: fetal calf serum
IFN: interferon
IL: interleukin
mAb: monoclonal antibody
MFI: mean fluorescence intensity
MG: Mitotracker green
miR: microRNA
MR: Mitotracker red
NCD4: naïve CD4 T cells
Ova: ovalbumin
PBMCs: peripheral blood mononuclear cells
ROS: reactive oxygen species
SOD: superoxide dismutase
TCRs: T cell receptors
WT: wild type
YNCD4: naïve CD4 T cells from young mice
