# Supplementary material for: Peripheral residence of naïve CD4 T cells induces MHC class II-dependent alterations in phenotype and function
Source: BMC Biol. 2014 Dec 21;12:106. doi: 10.1186/s12915-014-0106-0 (PMC4306244; doi:10.1186/s12915-014-0106-0)
Supplement: Additional file 1: Figure S1. — Additional figure providing NCD4lo and NCD4hi functional features. [file 12915_2014_106_MOESM1_ESM.docx]

Additional file 1.

Figure S1. NCD4lo cells respond poorly to activation as compared to NCD4hi cells even with IL-2 supplementation.


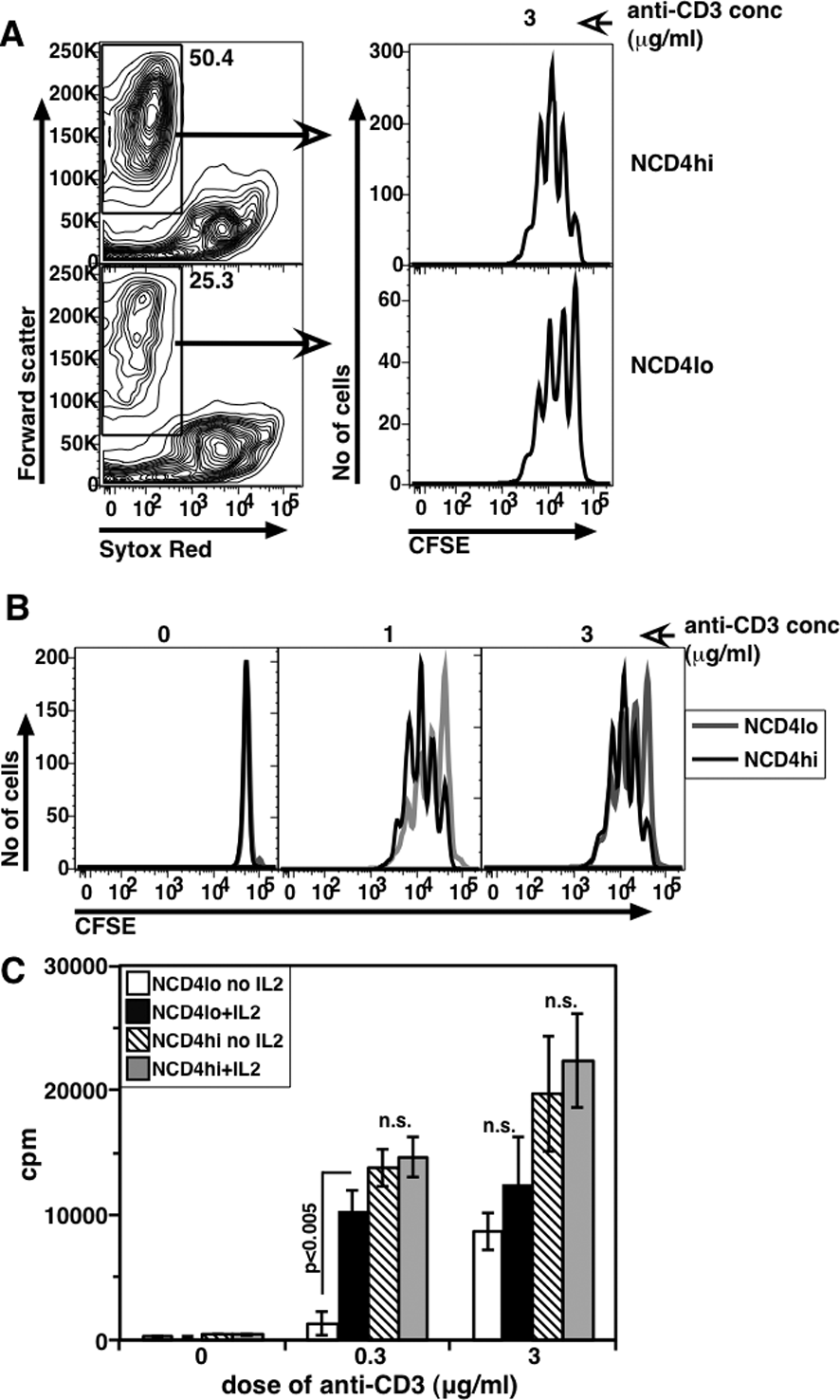


A. NCD4hi and NCD4lo cells were labeled with CFSE and activated with plate coated anti-CD3 in titrating doses and anti-CD28 (3μg/ml) for 60 h. Sytox red negative cells were gated as viable cells (left panels) and further analyzed for CFSE dilution profile.

B. Comparison of CFSE dilution curves of live gated NCD4hi and NCD4lo cells without activation (0 μg/ml anti-CD3, left) and for 2 doses (1 & 3 μg/ml) of anti-CD3 mediated activation. Data representative of 5 independent experiments.

C. 3H-thymidine incorporation by NCD4hi and NCD4lo cells in response to activation by anti-CD3 (titrating doses) and anti-CD28 (3 μg/ml), in presence or absence of exogenous IL-2. Data representative of 2 independent experiments.
