# Supplementary material for: Peripheral residence of naïve CD4 T cells induces MHC class II-dependent alterations in phenotype and function
Source: BMC Biol. 2014 Dec 21;12:106. doi: 10.1186/s12915-014-0106-0 (PMC4306244; doi:10.1186/s12915-014-0106-0)
Supplement: Additional file 2: Figure S2. — Additional figure providing NCD4lo and NCD4hi phenotypic and functional features. [file 12915_2014_106_MOESM2_ESM.docx]

Additional file 2

Figure S2. Additional phenotypic and functional features of NCD4hi and NCD4lo cells.


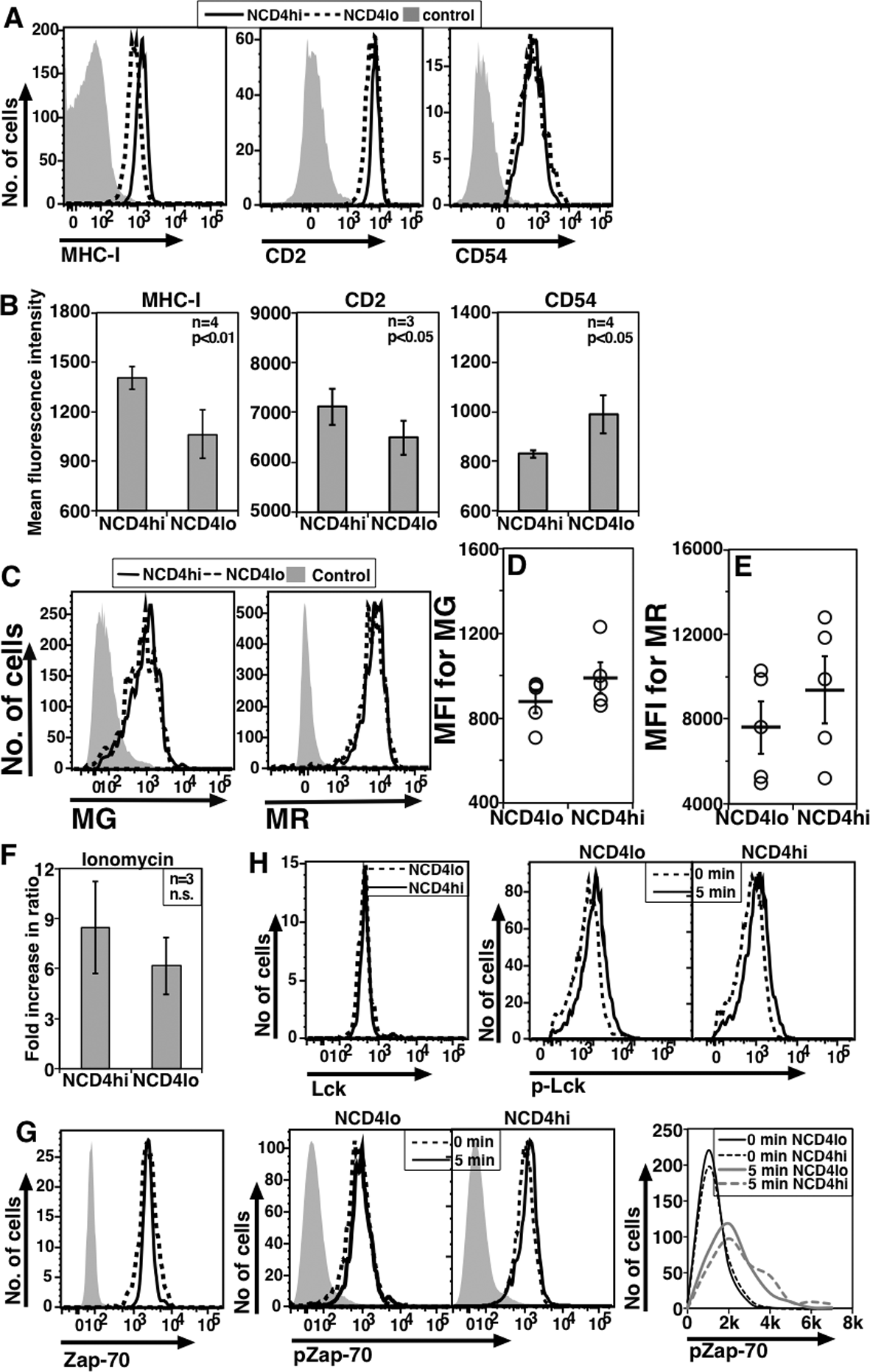


A. Representative staining for MHC-I, CD2 and CD54 on splenic NCD4hi and NCD4lo cells from young mice.

B. MFI values for MHC-I, CD2 and CD54 on NCD4hi and NCD4lo cells from multiple mice (mean + SE).

C. Representative histogram for MG (left panel) and MR (right panel) staining in NCD4lo and NCD4hi cells.

D. MFI for MG on NCD4hi and NCD4lo cells from 5 individual mice (mean + SE, 1 of 3 experiments).

E. MFI for MR on NCD4hi and NCD4lo cells from 5 individual mice (mean + SE, 1 of 3 experiments).

F. Fold increase in Fluo-3/Fura-Red ratio in NCD4hi and NCD4lo cells from independent mice following stimulation with ionomycin.

G. Representative staining for total Zap-70 in NCD4hi and NCD4lo cells (left). Comparison of pZap-70 levels at 0 and 5 min post-activation in NCD4lo and NCD4hi cells (middle and right, log and linear plots).

H. Representative staining for total Lck (left), p-Lck in NCD4lo (middle) and NCD4hi (right) cells before and 5 min post-activation with anti-CD3+anti-CD28.
