# Supplementary material for: Peripheral residence of naïve CD4 T cells induces MHC class II-dependent alterations in phenotype and function
Source: BMC Biol. 2014 Dec 21;12:106. doi: 10.1186/s12915-014-0106-0 (PMC4306244; doi:10.1186/s12915-014-0106-0)
Supplement: Additional file 3: Figure S3. — Additional figure providing data on NCD4 levels in an independent TCR-Tg mouse strain and shorter treatment of Euk-134 in vivo. [file 12915_2014_106_MOESM3_ESM.docx]

Additional File 3

Figure S3. Reduction in CD4 levels in KJ1.26+ DO11.10 cells parked in Balb.c mice and effect of short-term Euk-134 treatment in vivo.


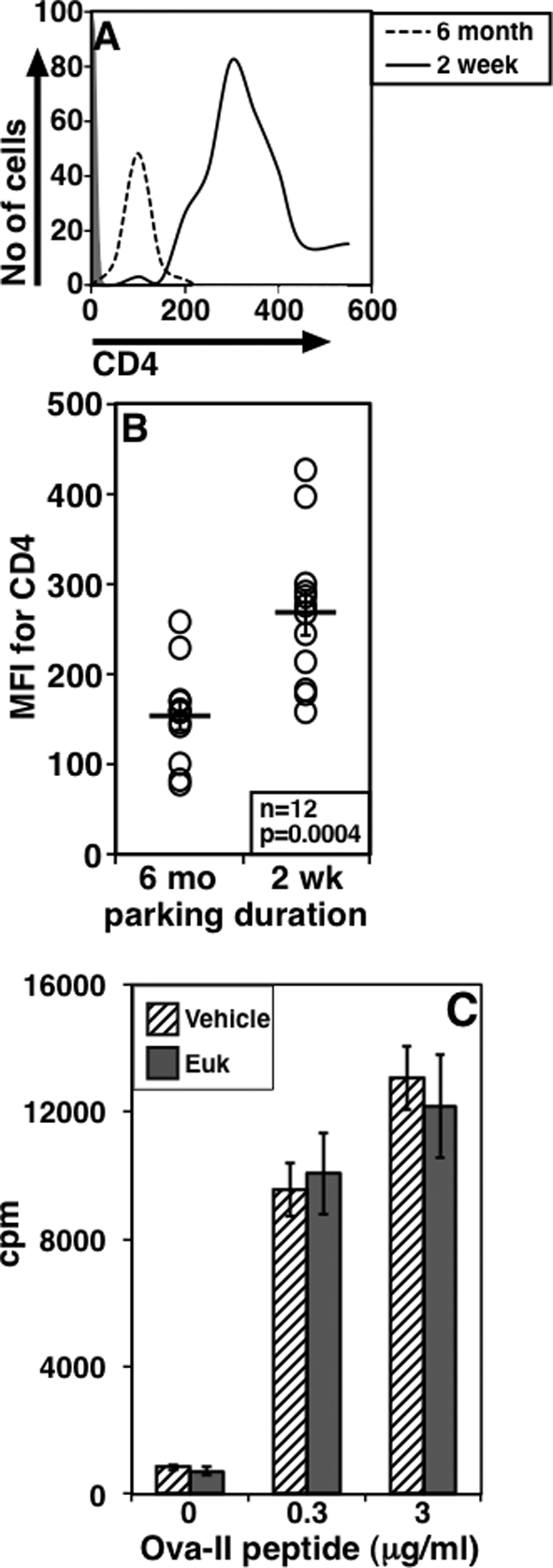


A. Comparison of CD4 levels on donor DO11.10 cells identified as CD4+KJ1.26+ cells in the spleens of recipient mice. Cells were transferred either 2 weeks or 6 months prior to euthanasia.

B. Pooled data from 12 mice in each category of parking plotted to show CD4 MFI values (Each circle represents one mouse, mean + SE shown).

C. 3H-thymidine incorporation in response to titrating concentrations of OVA-II peptide in OT-II cells parked for 2 weeks in WT mice (with or without Euk-134 treatment). Data normalized to 250 OT-II cells (Mean + SE of triplicate cultures; 1 of 2 experiments).
